# Structural and functional characterization of the newly identified *Photorhabdus laumondii* tumor necrosis factor‐like lectin

**DOI:** 10.1111/febs.70293

**Published:** 2025-10-17

**Authors:** Filip Melicher, Pavel Dobeš, Jan Komárek, Lukáš Faltinek, Marek Korsák, Petra Sýkorová, Josef Houser, Michaela Wimmerová

**Affiliations:** ^1^ Central European Institute of Technology (CEITEC), Masaryk University Brno Czech Republic; ^2^ National Centre for Biomolecular Research, Faculty of Science Masaryk University Brno Czech Republic; ^3^ Department of Experimental Biology, Faculty of Science Masaryk University Brno Czech Republic; ^4^ Department of Biochemistry, Faculty of Science Masaryk University Brno Czech Republic

**Keywords:** carbohydrate recognition, crystal structure, glycan binding, *Heterorhabditis bacteriophora*, Lectin, Lewis antigen, *Photorhabdus laumondii*, TNF fold

## Abstract

*Photorhabdus* bacteria live in mutualistic relationships with *Heterorhabditis* nematodes, and together, they act as effective insect pathogens. These bacteria produce a diverse array of lectins, sugar‐binding proteins that are believed to play crucial roles in the complex tripartite interaction among *Photorhabdus*, nematodes, and their insect hosts. One such lectin, *Photorhabdus laumondii* tumor necrosis factor (TNF)‐like lectin (PLTL), identified in *Photorhabdus laumondii* subsp. *laumondii* TTO1, exhibits notable sequence similarity to the N‐terminal domain of the BC2L‐C lectin (BC2L‐CN), a TNF‐like lectin recognized for its specificity toward fucosylated glycans associated with human embryonic stem cells and certain cancers. Through glycan array analysis and surface plasmon resonance, we identified PLTL's binding preference for branched histo‐blood group oligosaccharides. The crystallographic structure of PLTL in complex with the BLe^b^ pentasaccharide reveals a network of direct and water‐mediated hydrogen bonds simultaneously stabilizing the Fucα1‐2 and Galα1‐3 moieties, which define its narrow glycan specificity. A combination of mass spectrometry, protein crystallography, and analytical ultracentrifugation showed a unique hexameric PLTL architecture stabilized by intermolecular disulfide bridges. Our data suggest that PLTL may contribute to the mutualistic relationship between *Photorhabdus* and its nematode symbiont, *Heterorhabditis bacteriophora*, rather than playing a role in the interaction with the insect host. This study provides a structural and functional characterization of PLTL, a newly identified member of the TNF‐like lectin family. Comparative analysis with BC2L‐CN highlights both conserved and distinct structural features, suggesting potential applications in glycan recognition‐based diagnostics or biotechnological tools beyond its biological role. Our findings underscore its complex glycan specificity and offer insights into its potential role in *Photorhabdus*‐nematode symbiosis.

## Graphical abstract

We characterize the PLTL lectin from *Photorhabdus laumondii*, which adopts a unique TNF‐like fold. Binding assays reveal a preference for branched histo‐blood group oligosaccharides. Protein crystallography uncovers a novel hexameric assembly and identifies key residues involved in carbohydrate recognition. Biological data suggest that PLTL contributes to the mutualistic interaction between *P. laumondii* and *Heterorhabditis* nematodes.

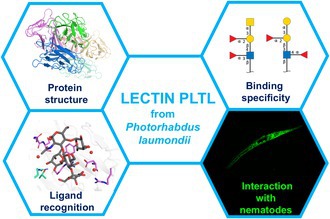

**Abbreviations**
ASU: asymmetric unit
AUC: analytical ultracentrifugation
BC2L‐CN: N‐terminal domain of *Burkholderia cenocepacia* lectin C
BGA: blood group A
BGB: blood group B
BGH: blood group H
BSA: bovine serum albumin
LC–MS/MS: Liquid Chromatography with tandem Mass Spectrometry
LRO: lysosome‐related organelles
MALDI: Matrix Assisted Laser Desorption/Ionization
nanoDSF: nano differential scanning fluorimetry
OD_600_
: optical density at 600 nm
PLTL: *Photorhabdus laumondii* tumor necrosis factor‐like lectin
RBC: red blood cell
RMSD: root mean square deviation
RSL: *Ralstonia solanacearum* lectin
SPR: Surface plasmon resonance
SDS/PAGE: sodium dodecyl‐sulfate polyacrylamide gel electrophoresis
TCEP: tris(2‐carboxyethyl)phosphine
TNF‐like: tumor necrosis factor‐like

## Introduction

The genus *Photorhabdus* represents a captivating group of Gram‐negative bioluminescent bacteria renowned for their highly specialized mutualistic relationship with entomopathogenic nematodes of the genus *Heterorhabditis* [1]. These nematodes traverse soil ecosystems searching for insect hosts, carrying the bacteria in the gut or specialized gut compartments. This symbiosis is mutually beneficial: the bacteria gain transport and protection from the nematodes during the initial stages of host infection, while the nematodes benefit from bacterial metabolites that play a significant role in the efficient killing and digestion of insect hosts, generating a nutrient‐rich environment for their development. This intricate relationship serves as a remarkable model for exploring host–microbe interactions and the co‐evolution of symbiotic partnerships [2, 3, 4]. Among the diverse array of substances produced by *Photorhabdus* spp. are lectins. These saccharide‐binding proteins mediate essential biological processes, including cell‐to‐cell communication and host‐pathogen recognition [5]. *Photorhabdus* species' genomes encode various lectins, which are hypothesized to play pivotal roles in the mutualistic and pathogenic stages of the entomopathogenic nematode–bacteria–insect interaction. Given their specificity and functional importance, *Photorhabdus* lectins have emerged as compelling targets for research [6, 7, 8, 9, 10, 11].

To date, representatives of two lectin families have been characterized in *Photorhabdus* species: the seven‐bladed β‐propeller lectin family includes PLL from *P. kayaii* [6], PHL from *P. asymbiotica* [7, 8], and five homologous lectins (PLL1–PLL5) from *P. laumondii* subsp. *laumondii* TTO1, which can bind to l‐fucose, d‐galactose, and *O*‐methylated saccharides [9, 11]. Another distinct lectin, PllA, belonging to the LecA‐like family, has been identified in *P. laumondii* and utilizes calcium ions to bind α‐*d*‐galactosylated glycans [10]. Although the biophysical properties, structures, and carbohydrate specificities of these lectins are well characterized, their biological roles remain to be uncovered.

Bioinformatic analysis of the *Photorhabdus laumondii* subsp. *laumondii* TTO1 genome has revealed another hypothetical lectin. This protein, named PLTL, shares a significant sequence identity (52%) with BC2L‐CN, from the opportunistic human pathogen *Burkholderia cenocepacia* [12]. BC2L‐CN is notable for its unique tumor necrosis factor‐like (TNF‐like) fold and narrow carbohydrate specificity for fucosylated glycans, particularly those with the Fucα1‐2Galβ1‐3GlcNAc/GalNAc motif (e.g., blood group H Type 1 epitope) [13]. Intriguingly, BC2L‐CN has demonstrated potential as a molecular probe for identifying human embryonic stem cells and undifferentiated human pluripotent stem cells [14, 15, 16]. Furthermore, BC2L‐CN has been studied as a potential system for targeted drug delivery [17, 18] due to its ability to recognize certain human cancer types, including breast, pancreatic, and prostate cancers [19, 20, 21]. These findings highlight the potential significance of PLTL in both biological research and biotechnological applications.

This study explores the structural and functional characteristics of PLTL, a TNF‐like lectin produced by *Photorhabdus laumondii* subsp. *laumondii* TTO1 and its role in the nematode–*Photorhabdus* symbiosis. By examining PLTL's unique features and its comparison with BC2L‐CN, this research aims to shed light on the broader biological significance of this lectin.

## Results

### Production and characterization of the PLTL

**Fig. 1:**
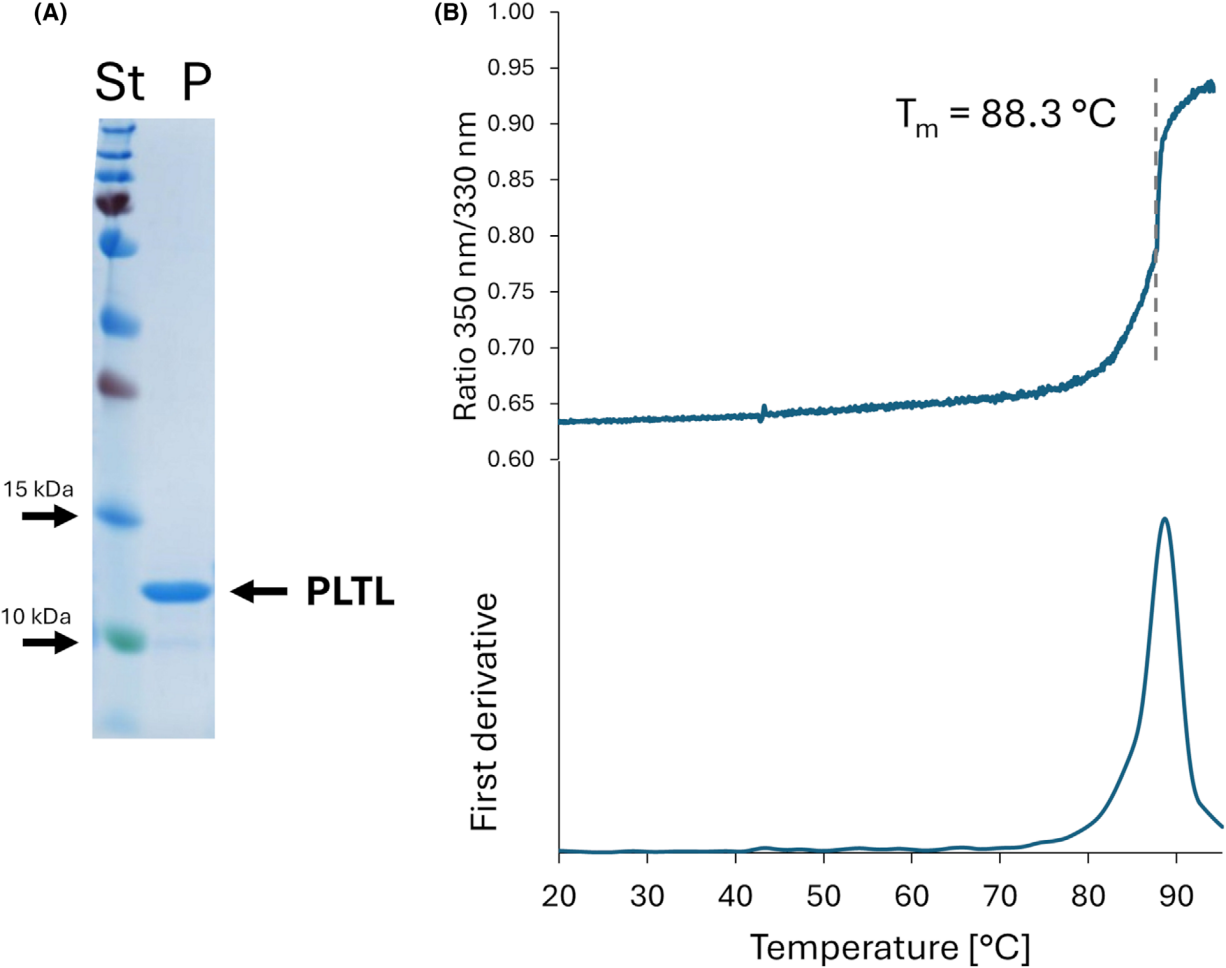
Purity and thermal stability of *Photorhabdus laumondii* tumor necrosis factor‐like lectin (PLTL). (A) Purity of the PLTL assessed by sodium dodecyl‐sulfate polyacrylamide gel electrophoresis (SDS/PAGE) electrophoresis. Protein ladder (St) and PLTL protein sample after the last purification step (P, 14.8 kDa) loaded on the 15% SDS/PAGE gel stained by Coomassie Brilliant Blue R‐250. (B) Thermal stability of the PLTL assessed by nanoDSF. The 350 nm/330 nm fluorescence ratio curve and its first derivative obtained during the experiment performed) in 100 mm bicine, pH 8.0, show PLTL *T*_m_ 88.3 °C at the inflection point (dashed line).

Native PLTL production in *Photorhabdus laumondii* subsp. *laumondii* TTO1 was assessed in minimal medium. LC–MS/MS analysis confirmed the production of wild‐type PLTL in the bacterial cytosol, though in only a small amount. Therefore, the PLTL lectin was produced in a recombinant form within the *Escherichia coli* expression system. Consequently, a multi‐step purification procedure involving heat denaturation of the protein contaminants, ammonium sulfate precipitation, and ion exchange chromatography was used to get PLTL in a pure form (Fig. 1A). Our protocol led to ~45 mg yield of PLTL per liter of the culture. PLTL was next characterized using a panel of biophysical techniques. The nanoDSF experiment revealed high thermal stability in the buffer 25 mm bicine, pH 8.0 (*T*_m_ = 88.3 °C, Fig. 1B).

**Fig. 2:**
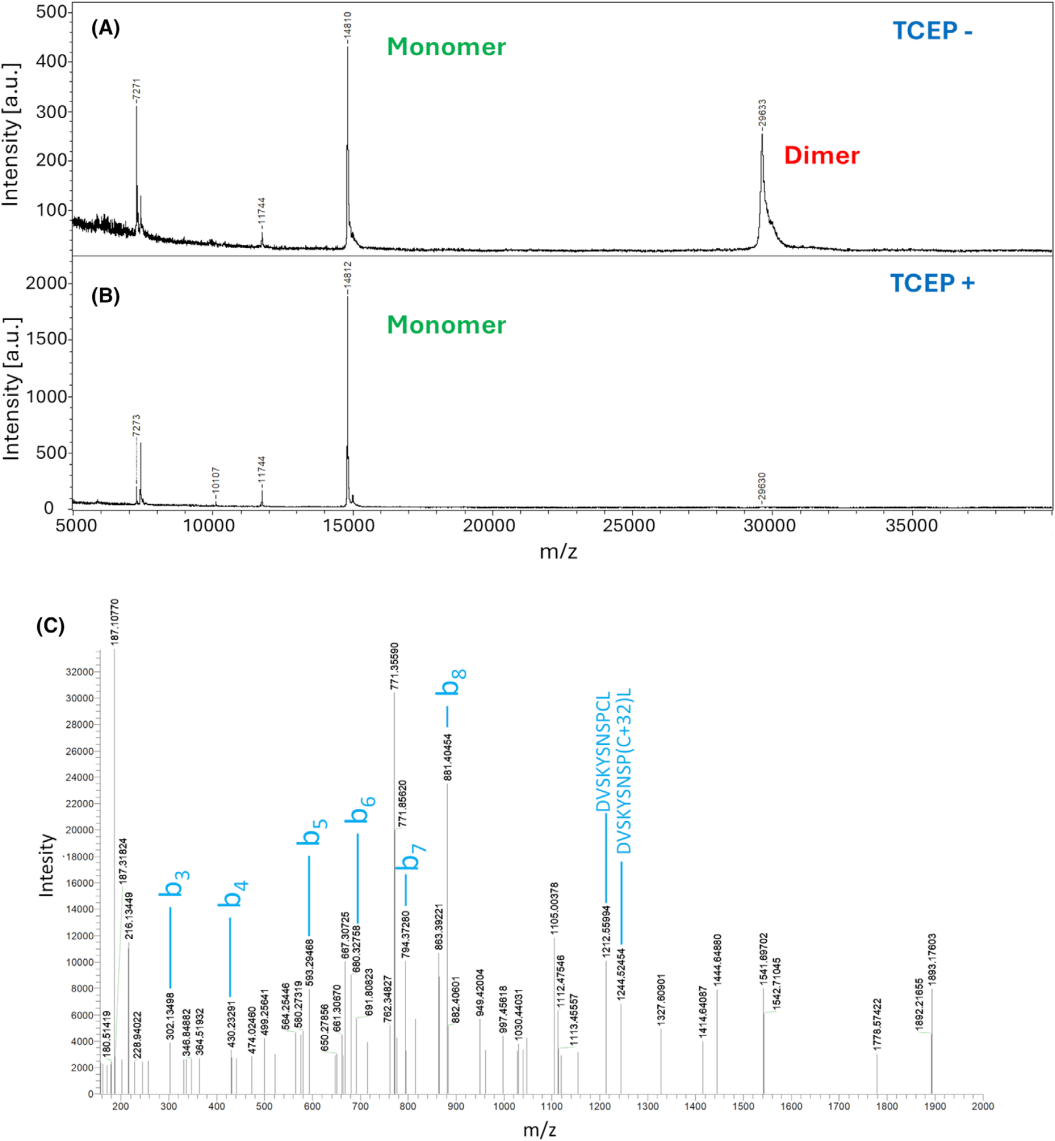
Mass spectrometry analyses of the *Photorhabdus laumondii* tumor necrosis factor‐like lectin (PLTL) lectin. (A) MALDI‐TOF intact mass analysis of PLTL without the reducing agent showed the presence of the monomer (14 810 Da) and covalently bound dimer (29 633 Da) in the sample. (B) MALDI‐TOF intact mass analysis of PLTL with 5 mm TCEP (tris(2‐carboxyethyl)phosphine) showed only the presence of the monomer (14 812 Da) in the sample. MALDI‐TOF mass spectra of the proteins were recorded on an Ultraflextreme instrument (Bruker Daltonics, Bremen, Germany) operated in the linear positive ion detection mode under the FlexControl 3.4 software (Bruker Daltonics). Mass spectra were processed with the FlexAnalysis 3.4 software (Bruker Daltonics). (C) LC–MS/MS analysis confirms the C40‐C40 disulfide formation. Results were obtained using the Orbitrap LUMOS instrument in HCD fragmentation mode. Peptide identity is confirmed based on characteristic signals—in addition to the standard C‐ and N‐terminal fragments (*b*_n_), the presence of a disulfide bond is verified by the fragmentation of the S‐S bridge, which is evidenced by the presence of fragment signals [M + H] + and [M + 32 + H]+, where M corresponds to free peptides. The proteins were subjected to proteolysis by pepsin (in 10 mm HCl for 1 h at 37 °C) prior to LC–MS/MS analyses. LC–MS/MS analyses of all peptide mixtures were done using RSLCnano system connected to Orbitrap Fusion Lumos Tribrid mass spectrometer (Thermo Fisher Scientific, Waltham, MA, USA). MS data were acquired in either data depended acquisition (DDA) or product ion scan mode with additional survey scan measurement (PRM). Orbitrap analyzer resolution of 30 000 was used for all MS/MS spectra.

The intact mass of PLTL was determined by MALDI‐MS. In the absence of a reducing agent, two species were detected in the spectrum: 14810 and 29 627 Da, indicating the presence of covalently linked dimers (Fig. 2A). Upon reduction, a single species with a molecular weight of 14 812 Da was observed (Fig. 2B), matching the theoretical mass of the PLTL monomer after cleavage of the initial methionine (14 811 Da). Subsequent LC–MS/MS analysis confirmed the presence of intermolecular disulfide bonds formed between Cys40 residues (Fig. 2C).

**Fig. 3:**
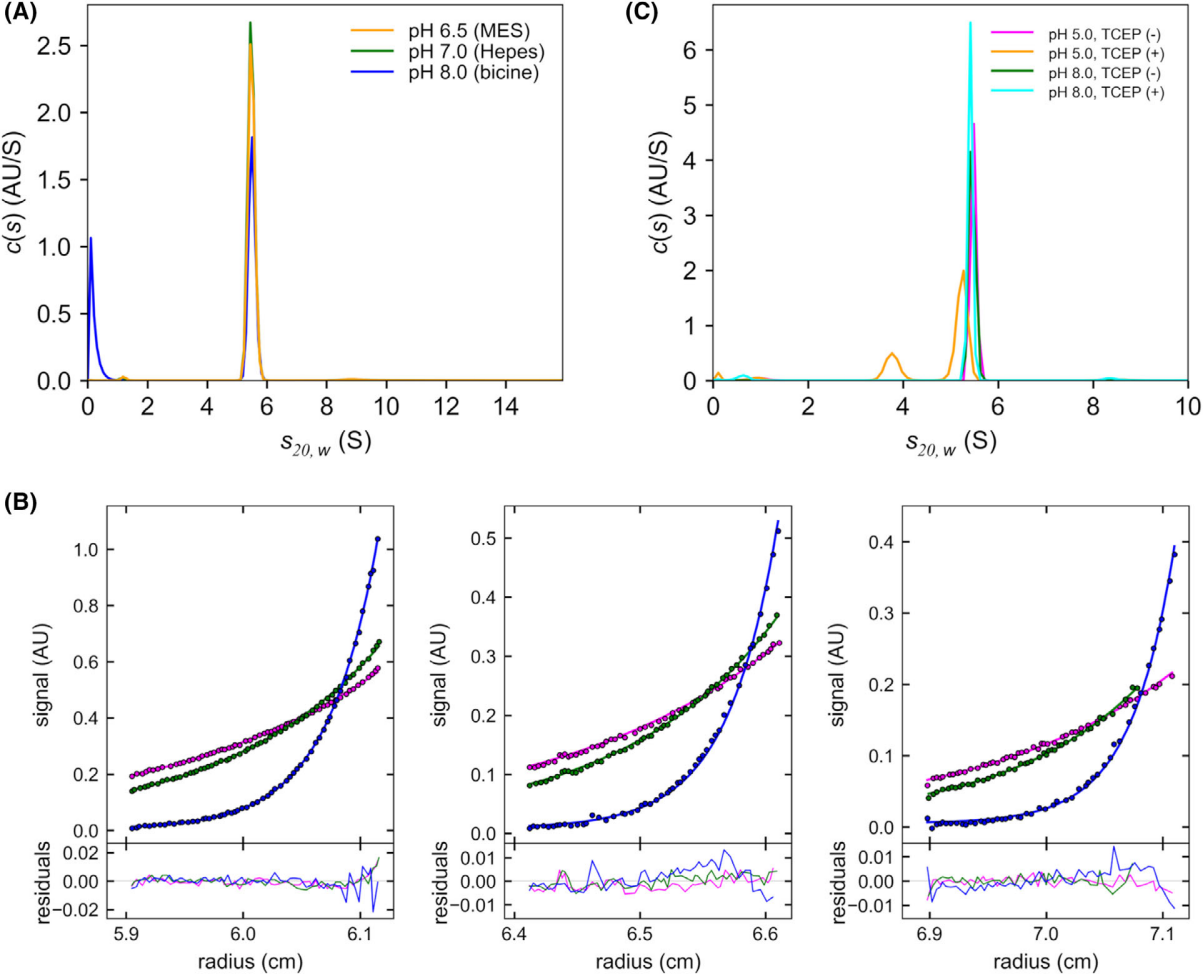
Oligomeric state and homogeneity of *Photorhabdus laumondii* tumor necrosis factor‐like lectin (PLTL) assessed by analytical ultracentrifugation. (A) Homogeneity and oligomeric state of PLTL in various solvents studied by sedimentation velocity experiment. The measurement was performed at 20 °C and 48 000 rpm with 0.62 mg·mL^−1^ PLTL in (i) 25 mm MES, 150 mm NaCl, pH 6.5, (ii) 25 mm HEPES, 150 mm NaCl, pH 7.0, and (iii) 30 mm bicine, pH 8.0, respectively. The c(s) analysis shows homogenous distributions with the predominant peak corresponding to PLTL hexamer (5.5 S). Please note that the c(s) distributions were corrected for different densities and viscosities of the solvents (sedimentation coefficients at standard conditions are shown). (B) Determination of PLTL molar mass by the sedimentation equilibrium (SE) technique. SE profiles were obtained at 20 °C at rotor speeds of 9000 rpm (magenta), 10 800 rpm (green), and 19 000 rpm (blue) by measuring the absorbance (280 nm) of 0.35 mg·mL^−1^ (left panel), 0.17 mg·mL^−1^ (middle panel) and 0.09 mg·mL^−1^ PLTL (right panel) in 25 mm MES, 150 mm NaCl, pH 6.5. The global fitting of SE data provided a value of 88.0 kDa, which perfectly agrees with the molar mass of PLTL hexamer (88.9 kDa based on protein sequence). The residual plots show the goodness of the fit. (C) The effect of reducing agent and pH on the oligomeric state of PLTL. Sedimentation velocity experiment was performed at 20 °C and 48 000 rpm with 0.62 mg·ml^−1^ PLTL in 25 mm Na citrate, 150 mm NaCl, pH 5.0, or 25 mm bicine, 150 mm NaCl, pH 8.0 buffers, both with and without 5 mm TCEP (tris(2‐carboxyethyl)phosphine).

In order to determine the oligomeric state of PLTL in the solution, analytical ultracentrifugation was performed. The sedimentation velocity experiment showed that the sedimentation coefficient (s_20,w_) of PLTL is 5.5 S, which corresponds to the PLTL hexamer, and the determined frictional ratio (*f*/*f*_0_ = 1.3) suggested a moderately elongated shape of the molecule. The PLTL hexamer was stable in various buffers with pH ranging between 5.0 and 8.0 (Fig. 3A), and the sample showed homogeneous distributions in all tested solvents. The presence of PLTL in a hexameric form was further confirmed by the sedimentation equilibrium technique (Fig. 3B). The determined molecular weight of PLTL was 88.0 kDa, which is in excellent agreement with the theoretical value of the PLTL hexamer (88.9 kDa, calculated from protein sequence). An additional sedimentation velocity experiment was performed to investigate the effect of the pH and presence of the reducing agent TCEP (Tris(2‐carboxyethyl)phosphine) on the PLTL oligomeric stability. At pH 8.0, PLTL was present in the hexameric form (s_20,w_ = 5.5 S), regardless of TCEP presence. At pH 5.0, PLTL maintained its hexameric structure under acidic conditions when TCEP was not present but partially dissociated into trimers (s_20,w_ = 3.8 S) in the presence of the reducing agent (Fig. 3C).

### PLTL carbohydrate specificity

**Fig. 4:**
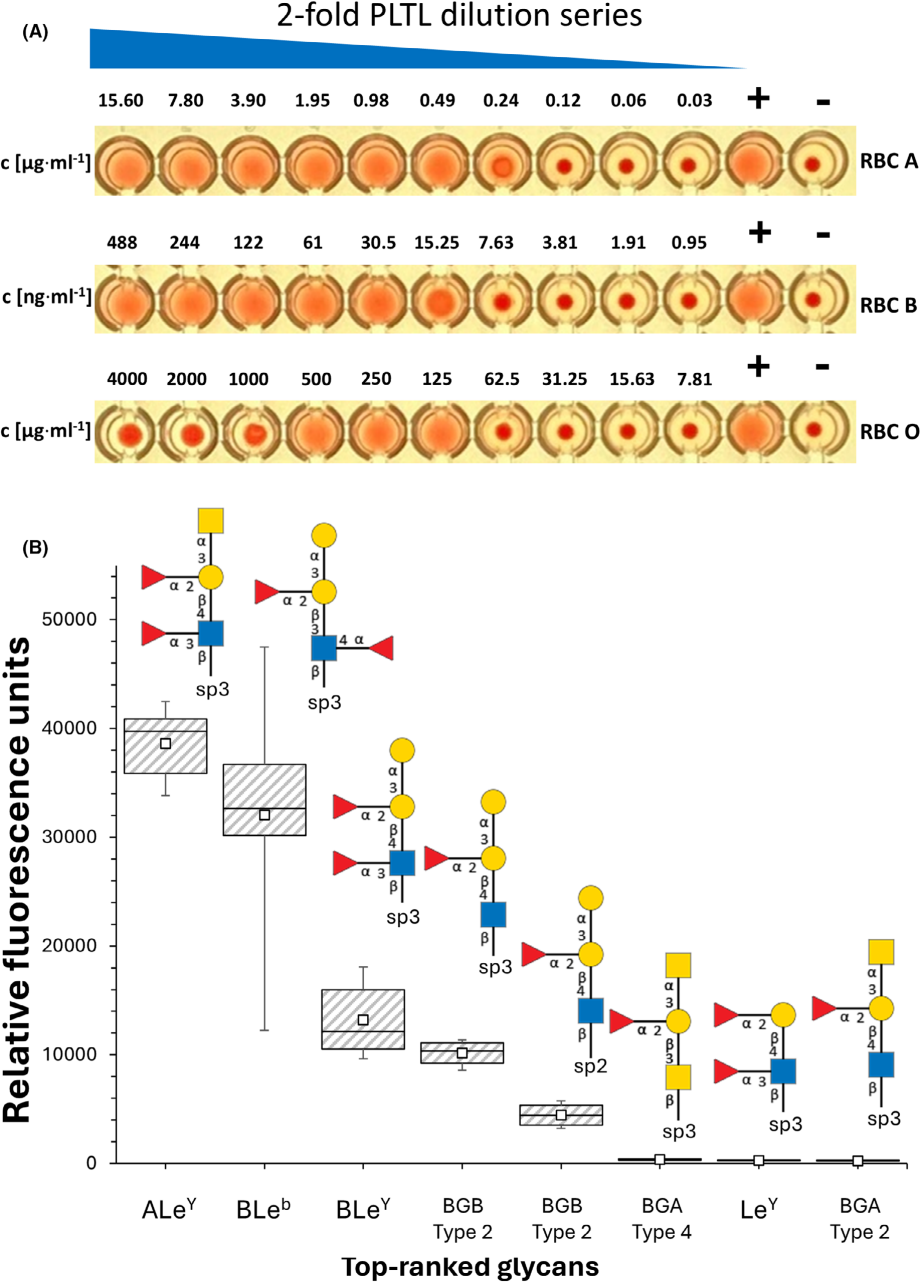
Carbohydrate‐binding properties of *Photorhabdus laumondii* tumor necrosis factor‐like lectin (PLTL). (A) Hemagglutination assay with red blood cells (RBC) groups A, B, and O performed in a microplate as a twofold dilution series with decreasing lectin concentration from left to right. The strongest hemagglutination was observed with the blood group B RBC, where the minimal hemagglutination concentration (MHC) was 30.5 ng·mL^−1^. The observed MHC for blood group A and O were 490 ng·mL^−1^ (one order higher) and 125 μg·mL^−1^ (four orders higher), respectively. The wells (+) column represents the positive control (250 μg·mL^−1^ RSL), and the wells in the (−) column represent the negative control (PBS buffer). Agglutinated red blood cells form a diffuse mat, whereas nonagglutinated red blood cells sediment and form a clear dot at the bottom of the well. ‘Super‐agglutinated’ red blood cells form a large precipitate (first three wells in the third row). (B) Box and whisker plot for glycan array screening of DyLight488‐labeled PLTL (*n* = 6). Depiction of the eight oligosaccharides with a signal‐to‐noise ratio greater than 10. Formulas for linkers: sp2, ‐O‐(*p*‐C_6_H_4_)‐O‐CH_2_CH_2_NH_2_; sp3, ‐O‐(CH_2_)_3_NH_2_. The box's lower and upper edges represent the first and third quartiles, with the band inside indicating the median. The whiskers extend to the minimum and maximum values of the data, and the small squares within the boxes signify the mean. Glycan residues are represented according to the Symbol Nomenclature for Glycans guidelines [52].

A hemagglutination assay was employed to verify the activity of the purified PLTL lectin. PLTL was active, agglutinating all tested red blood cells (RBC) (human blood groups A, B, and O), however, with notable differences (Fig. 4A). The strongest hemagglutination was observed with the blood group B RBCs, where the minimal hemagglutination concentration (MHC) was 30.5 ng·mL^−1^. The observed MHC for blood group A and O was 490 ng·mL^−1^ (one order higher) and 125 μg·mL^−1^ (four orders higher), respectively. Although all human RBC antigens of the ABO system are fucosylated, our findings suggest a rather narrow carbohydrate specificity of PLTL.

A glycan microarray analysis was performed to elucidate the fine carbohydrate specificity of PLTL (Fig. 4B). Of the 381 glycoconjugates present, PLTL recognized only a specific subset of fucosylated glycans. The protein bound branched fucosylated oligosaccharides of the Lewis human histo‐blood group system, namely ALe^Y^ pentasaccharide (GalNAcα1‐3(Fucα1‐2)Galβ1‐4(Fucα1‐3)GlcNAcβ), BLe^Y^ pentasaccharide (Galα1‐3(Fucα1‐2)Galβ1‐4(Fucα1‐3)GlcNAcβ), BLe^b^ pentasaccharide (Galα1‐3(Fucα1‐2)Galβ1‐3(Fucα1‐4)GlcNAcβ), and blood group B (BGB) Type 2 tetrasaccharide (Galα1‐3(Fucα1‐2)Galβ1‐4GlcNAcβ). Other glycans with low overall signal but with signal‐to‐noise ratio greater than 10 included blood group B Type 4 tetrasaccharide (Galα1‐3(Fucα1‐2)Galβ1‐3GalNAcβ), Le^Y^ tetrasaccharide (Fucα1‐2Galβ1‐4(Fucα1‐3)GlcNAcβ), and blood group A (BGA) Type 4 tetrasaccharide (GalNAcα1‐3(Fucα1‐2)Galβ1‐4GlcNAcβ). Notably, despite being represented on the chip, no binding was observed to other human histo‐blood group oligosaccharides, including Le^a^, Le^X^, and Le^b^, blood group H antigens, as well as their modified version SiaLe^X^ and SiaLe^a^.

**Fig. 5:**
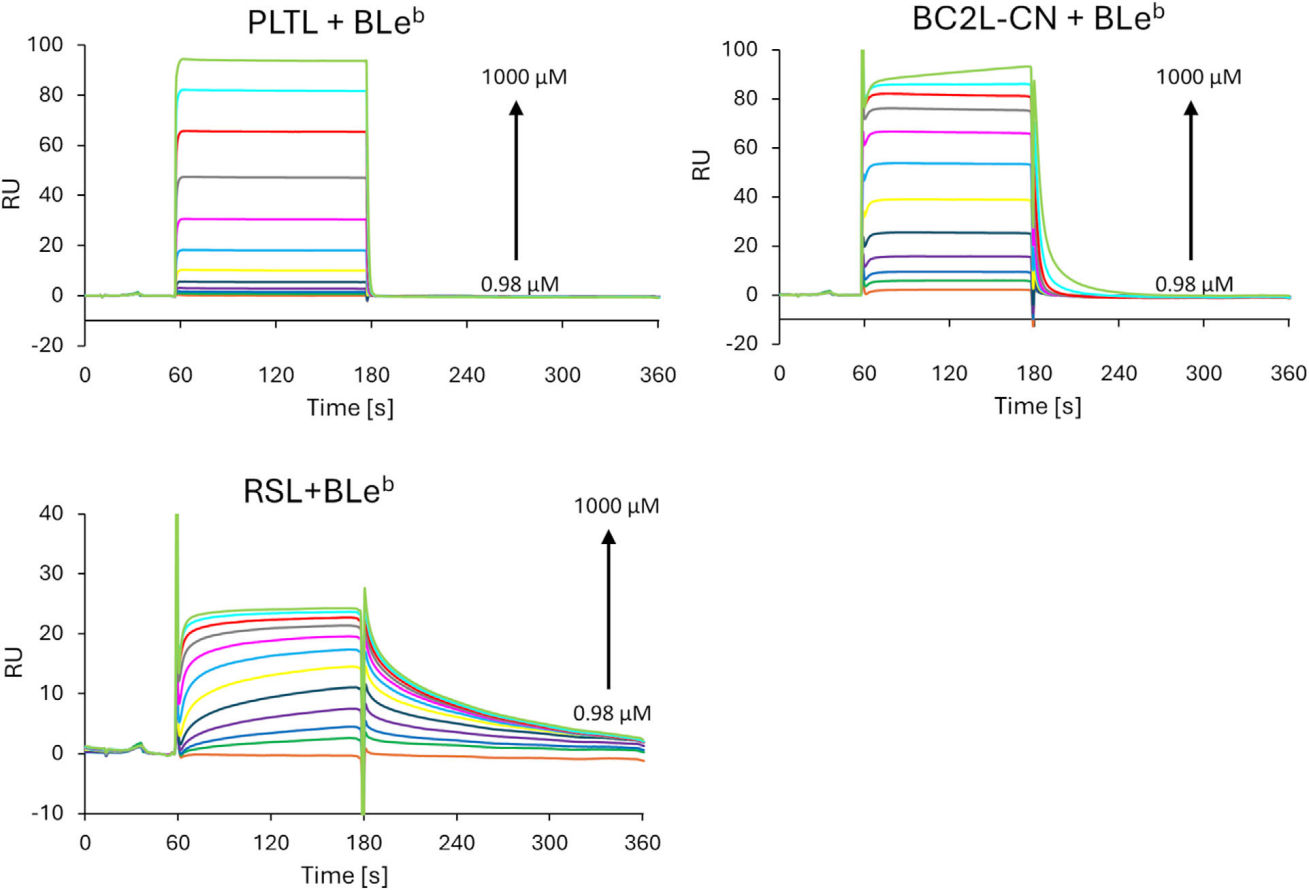
Surface plasmon resonance (SPR) sensorgrams displaying the BLe^b^ pentasaccharide binding to lectins *Photorhabdus laumondii* tumor necrosis factor‐like lectin (PLTL), BC2L‐CN, and RSL (*n* = 3). Response increases with increasing concentrations of the pentasaccharide (two‐fold dilution series from 1000 μm to 0.98 μm).

In the next step, the surface plasmon resonance (SPR) method was used to determine the equilibrium dissociation constant (*K*_D_) of the interaction between immobilized PLTL and various monosaccharides (l‐fucose, d‐mannose, *N*‐acetyl‐*d*‐galactosamine, d‐galactose) and fucose‐containing oligosaccharides. For comparison, two other fucose‐binding lectins (RSL from *R. solanacearum* [22] and the homologous BC2L‐CN lectin from *B. cenocepacia* [12]) were included in the experiment (Fig. 5 and Figs S1–S3). The determined apparent *K*_D_ values are summarized in Table 1. Among the tested monosaccharides, PLTL exhibited binding solely to l‐fucose, however, with low affinity (*K*_D_ ~ 3.6 mm). Regarding human blood group trisaccharides, PLTL displayed the highest affinity for the BGB trisaccharide (Galα1‐3(Fucα1‐2)Galβ), followed by the BGA trisaccharide (GalNAcα1‐3(Fucα1‐2)Galβ), and the lowest affinity for the BGH Type 2 trisaccharide (Fucα1‐2Galβ1‐4GlcNAcβ). These findings align with the previous results obtained from the hemagglutination assay. For Lewis type 1 antigens, PLTL's affinity increased with the oligosaccharides' size. The Le^a^ trisaccharide showed the lowest affinity, followed by the Le^b^ tetrasaccharide, while the highest affinity was observed for the BLe^b^ pentasaccharide.

**Table 1:** Determination of apparent equilibrium dissociation constants (*K*_D_) by SPR for the interaction between tested l‐fucose‐containing saccharides and the PLTL, BC2L‐CN, and RSL lectins (n = 3). Values in brackets refer to the determined *K*_D_ in previous studies for the BC2L‐CN [12] and the RSL [22]. The term ‘NB’ (no binding) is used for cases where no measurable interaction was detected under the experimental conditions.

	PLTL	BC2L‐CN	RSL
l‐Fucose	3.66 ± 0.18 mm	NB	1.74 ± 0.51 μm
Fuc(1,2)Gal	5.12 ± 3.28 mm *	NB	1.13 ± 0.53 μm (0.59 μm)
Fuc(1,4)GlcNAc	1.77 ± 0.70 mm *	3.72 ± 3.61 mm *	1.27 ± 0.21 μm
BGB trisaccharide	1.15 ± 0.26 mm *	NB	0.83 ± 0.16 μm (0.21 μm)
BGA trisaccharide	2.18 ± 0.25 mm *	2.06 ± 0.99 mm *	0.89 ± 0.28 μm (0.25 μm)
BGH type 2 trisaccharide	4.69 ± 1.23 mm *	942 ± 178 μm	0.95 ± 0.20 μm (0.82 μm)
Le^X^	NB	1.45 ± 0.59 mm *	22.0 ± 0.47 μm (14.4 μm)
Le^Y^	872 ± 132 μm	48 ± 5.6 μm (52.6 μm)	2.06 ± 0.01 μm
Le^a^	4.69 ± 1.71 mm *	368 ± 159 μm	5.90 ± 0.73 μm (3.71 μm)
Le^b^	559 ± 3.80 μm	1.91 ± 0.13 μm	4.55 ± 0.44 μm
BLe^b^	144 ± 32.8 μm	23.2 ± 2.6 μm	10.1 ± 1.6 μm

* Values estimated from the binding curves, where *K*_D_ is higher than the highest concentration of the ligand.

Regarding Lewis type 2 antigens, PLTL demonstrated sub‐millimolar affinity for the Le^Y^ tetrasaccharide while not interacting with the Le^X^ trisaccharide. These results suggest that Lewis type 2 oligosaccharides are recognized through Fucα1‐2, and the Fucα1‐3 moiety is not the preferred epitope. Unlike PLTL, BC2L‐CN bound to the BGH Type 2 trisaccharide with sub‐millimolar affinity and exhibited higher affinities for all Lewis antigens. Interestingly, while the branching of the BLe^b^ pentasaccharide led to a weaker interaction compared with the Le^b^ tetrasaccharide, PLTL showed the opposite trend, with a stronger affinity for BLe^b^. Among the tested lectins, RSL displayed the highest *K*_D_ toward all fucose‐containing saccharides, exhibiting a preference toward blood group trisaccharides. Notably, the binding kinetics of the RSL demonstrated distinct differences across all tested ligands and consistently showed slower association and dissociation rates compared with PLTL and BC2L‐CN lectins.

### Overall PLTL structure

Protein X‐ray crystallography was utilized to determine the structure of PLTL and revealed molecular details of its glycan recognition. PLTL formed distinct crystal morphologies across various crystallization conditions and ligands; nevertheless, all crystals diffracted at high resolution (1.2–1.6 Å). Detailed data collection and refinement statistics for all structures are shown in Table 2.

**Table 2:** Details of data and refinement statistics.

	apo‐PLTL	PLTL/BGB	PLTL/Le^Y^	PLTL/BLe^b^
PDB ID	9IB6	9IB7	9IB8	9IB9
Beamline	BESSY II 14.1	BESSY II 14.1	PETRA III P13	PETRA III P13
Wavelength (Å)	0.9763	0.9763	0.9763	0.9763
Space group	*H*32	*P*4_1_2_1_2	*P*4_1_2_1_2	*P*12_1_1
Unit‐cell parameters				
*a* (Å)	83.50	81.82	81.85	50.30
*b* (Å)	83.50	81.82	81.85	166.98
*c* (Å)	105.58	112.78	112.57	50.35
*α*/*β*/*γ* (°)	90, 90, 120	90, 90, 90	90, 90, 90	90, 106.60, 90
Resolution range (Å)	42.67–1.20 (1.26–1.20)	46.47–1.50 (1.58–1.50)	46.38–1.60 (1.69–1.60)	48.24–1.30 (1.37–1.30)
No. of unique reflections	44 331 (6417)	61 937 (8898)	51 136 (7323)	186 694 (26429)
< I/s(I) >	18.9 (1.7)	25.5 (2.7)	27.7 (3.7)	12.6 (2.0)
CC_1/2_	1.0 (0.84)	1.0 (0.85)	1.0 (0.89)	0.99 (0.69)
Wilson B factor (Å^2^)	10.1	19.5	22.8	13.9
Completeness (%)	100.0 (100.0)	100.0 (100.0)	100.0 (100.0)	96.2 (93.4)
*R*_merge_	0.098 (2.385)	0.077 (1.433)	0.069 (1.115)	0.065 (0.844)
Multiplicity	19.7 (19.5)	26.0 (24.9)	26.3 (26.8)	7.1 (6.9)
*R*_work_	0.154	0.174	0.189	0.150
*R*_free_	0.179	0.199	0.208	0.180
No. of protomers in ASU	1	3	3	6
RMS (bonds) (Å)	0.012	0.014	0.010	0.010
RMS (angles) (°)	1.794	1.766	1.806	1.697
Average B factor (Å^2^)	17.18	22.86	25.01	18.41
Protein	15.91	22.01	24.57	17.57
Water	31.1	31.34	28.80	26.32
Ligand	‐	27.95	34.63	21.48
Ramachandran plot profile (%)				
Favored	97	97	98	97
Allowed	3	3	2	3
Disallowed	0	0	0	0

**Fig. 6:**
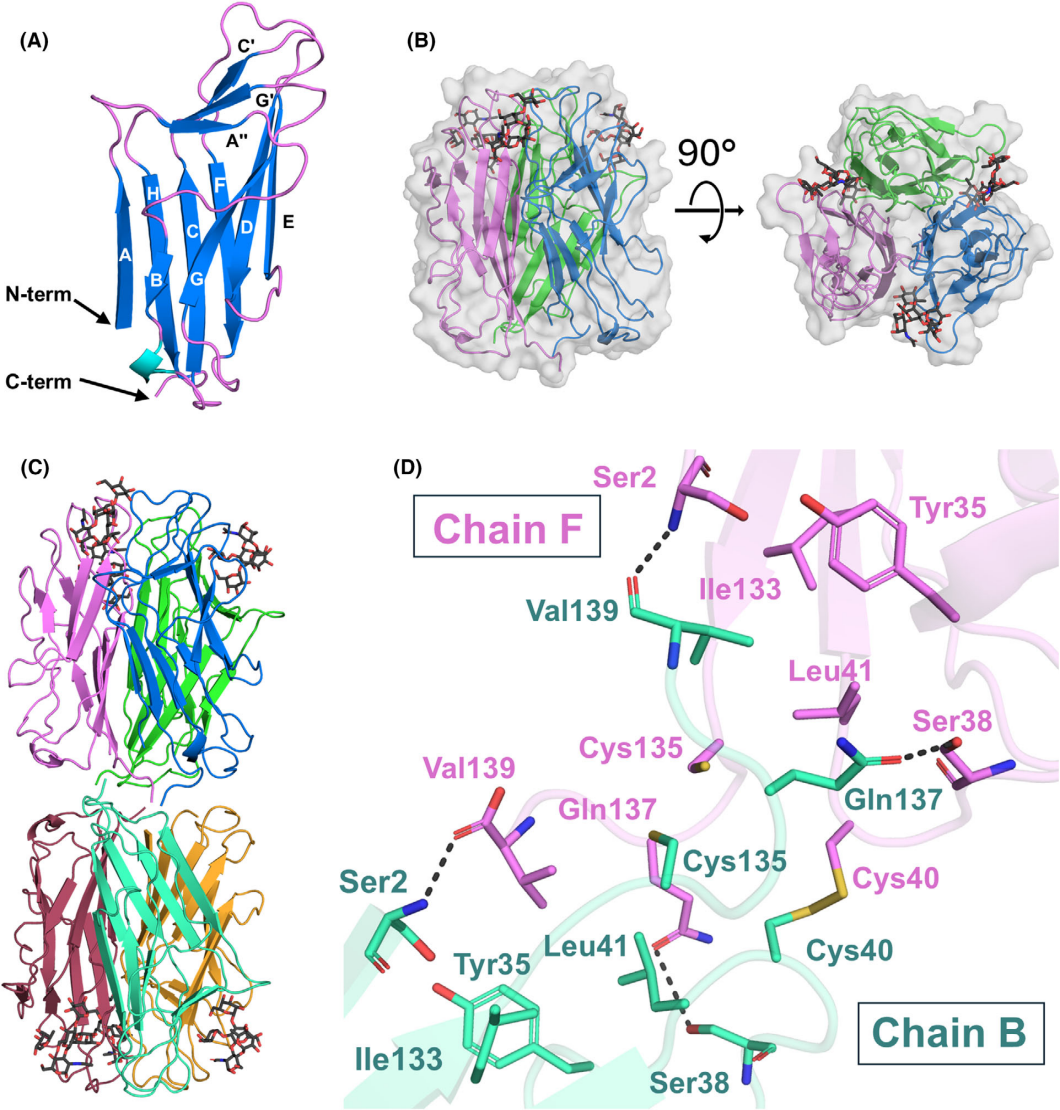
Crystal structure of the *Photorhabdus laumondii* tumor necrosis factor‐like lectin (PLTL) lectin. (A) Ribbon representation of the apo‐PLTL protomer (PDB: 9IB6) with highlighted β‐strands. (B) A TNF‐like structure formed by the PLTL trimer. (C) Architecture of the PLTL hexamer in complex with BLe^b^ (represented as black sticks, PDB: 9IB9). (D) Interaction interface between opposing chain B (green) and chain F (violet) (PDB: 9IB9). Amino acids involved in hexameric formation are represented as sticks, and polar contacts are represented as black dashed lines. All molecular structure images were generated using Pymol 3.0 (Schrödinger, LLC).

Each PLTL protomer consists of 11 β‐strands that were named according to the convention for TNF‐like proteins [12, 23] as A, A″, B, C, C′, D, E, F, G, G′, and H. β‐strands are connected by flexible loops and arranged in a Greek key topology, forming the overall jelly roll fold (Fig. 6A). The protein N terminus has a well‐defined electron density in all protomers within the asymmetric unit (ASU) and corresponds to Ser2. This finding agrees with the previous MS intact mass analysis, which showed that the initial methionine was cleaved off. The C‐terminal Val139 is resolved only in the PLTL/BLe^b^ complex for chains B, E, and F. In all other structures, the C termini are positioned along the crystallographic axes, where their electron density remains poorly defined.

Three PLTL protomers are symmetrically arranged around a noncrystallographic threefold axis, resembling a typical trimeric TNF‐like structure (Fig. 6B), stabilized by hydrogen bonds and hydrophobic interactions. The crystallographic structure of PLTL consistently shows a hexameric oligomeric state across all resolved structures, which agrees with the analytical ultracentrifugation analysis. The PLTL is unique in its hexameric architecture formed by two TNF‐like structures interacting by opposing N‐ and C termini (Fig. 6C). The interaction interface is established through the intertwining of opposing protomers, stabilized by hydrogen bonds between Gln137 and Ser38, as well as the carboxyl group of the C‐terminal Val139 bonding with the amino group of N‐terminal Ser2. In addition, the Val139 side chain interacts with a hydrophobic pocket formed by Tyr35, Leu41, and Ile133 of the opposing protomer (Fig. 6D). Interestingly, the C termini were well defined only in three out of six protomers (only visible in PLTL/BLe^b^ complex), which suggests that the interaction interface is structurally flexible. Furthermore, the interaction of two opposing monomers is covalently stabilized through an intermolecular C40–C40 disulfide bridge, consistent with our mass spectrometry analysis.

### Structural basis of carbohydrate recognition

**Fig. 7:**
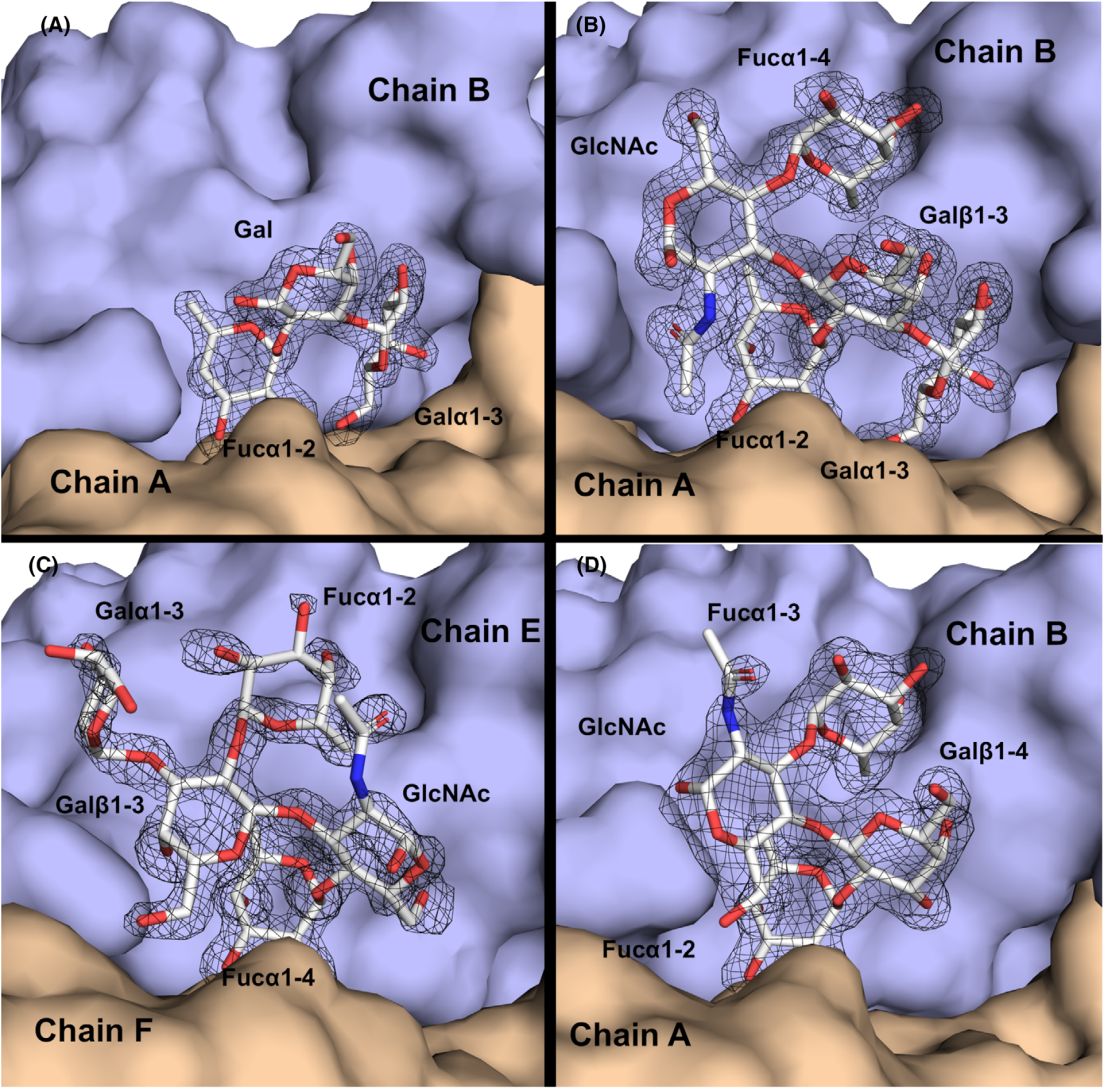
Surface representation of the binding interface between protomers with Fo‐Fc electron density at 3σ. Electron density is displayed around the BGB trisaccharide (A), the BLe^b^ pentasaccharide in canonical orientation (B), the BLe^b^ pentasaccharide in noncanonical orientation (C), and the Le^Y^ tetrasaccharide (D). All molecular structure images were generated using Pymol 3.0 (Schrödinger, LLC).

PLTL was successfully co‐crystallized with BGB trisaccharide (Galα1‐3(Fucα1‐2)Galβ1‐3), Le^Y^ tetrasaccharide (Fucα1‐2Galβ1‐4(Fucα1‐3)GlcNAcβ), and BLe^b^ pentasaccharide (Galα1‐3(Fucα1‐2)Galβ1‐3(Fucα1‐4)GlcNAcβ). A closer inspection of the Fo‐Fc electron density from all datasets revealed a well‐defined electron density of the corresponding oligosaccharides (Fig. 7). The carbohydrate‐binding pocket is situated at the interface between two adjacent protomers on either end of the hexamer, with a total of six binding sites. Ligands are stabilized through hydrophobic interactions and a complex network of direct and water‐mediated hydrogen bonds from both protomers (Table S1).

**Fig. 8:**
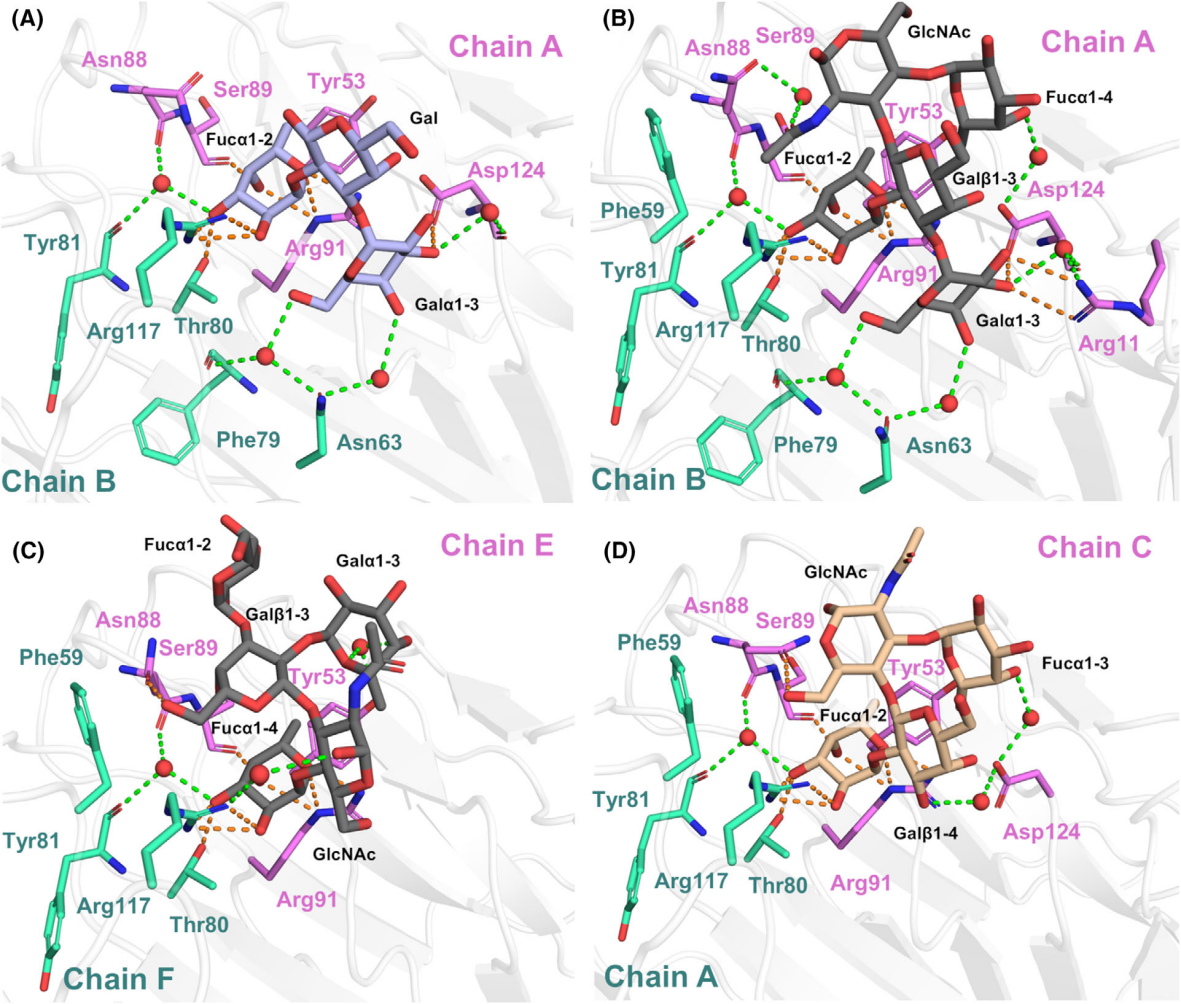
*Photorhabdus laumondii* tumor necrosis factor‐like lectin (PLTL) binding site interactions with various saccharides in the crystal structures. (A) The BGB trisaccharide (light blue sticks) and (B) the BLe^b^ pentasaccharide (gray sticks) in the canonical saccharide orientation. (C) The BLe^b^ in a noncanonical binding orientation and (D) the Le^Y^ tetrasaccharide (beige sticks). Amino acids involved in saccharide interactions are shown as green and violet sticks. Direct polar contacts between the protein and saccharide are depicted by orange dashed lines, while polar contacts mediated via water molecules (red spheres) are indicated by green dashed lines. All molecular structure images were generated using Pymol 3.0 (Schrödinger, LLC).

The crystal structure of the PLTL/BGB complex was determined at a resolution of 1.5 Å. The complex crystallized in the *P*4_1_2_1_2 space group with three protomers in the ASU. A well‐defined electron density for the trisaccharide was observed between chains A and B (Fig. 8A), as well as between chains A and C. In contrast, the BGB trisaccharide located in the binding site between chains B and C is more exposed to the solvent, with clear electron density only for the Fucα1‐2 and Galα1‐3 moieties. A detailed analysis of the binding site revealed that Fucα1‐2 is primarily stabilized through direct hydrogen bonds with Thr80 and Arg117 from one protomer and Ser89 and Arg91 from the neighboring protomer. Additionally, a single water‐mediated hydrogen bond is present between the oxygen atom O3 and the carbonyl groups of Asn88 and Tyr81. The C6 methyl group of Fucα1‐2 engages in a hydrophobic CH‐π stacking interaction with the aromatic ring of Tyr53. In contrast, Galα1‐3 forms only one direct interaction, where its oxygen atom O3 establishes a hydrogen bond with the side chain of Asp124. Additional stabilization of Galα1‐3 occurs through three water‐mediated hydrogen bonds, involving atoms O3, O4, and O6, and three water molecules. A key stabilizing factor for the BGB trisaccharide is Arg91, which simultaneously interacts with both O5 and O4 of Fucα1‐2 and participates in a hydrophobic interaction with the sugar ring of the Galα1‐3 moiety.

The PLTL/BLe^b^ complex crystallized in the *P*12_1_1 space group, and its structure was refined to a resolution of 1.3 Å. With six protomers in the ASU, the entire ligand could be reliably modeled in all six possible binding sites. Two distinct ligand‐binding orientations were observed for the BLe^b^ pentasaccharide. The canonical orientation (Fig. 8B), previously observed in the homologous protein BC2L‐CN [12, 13], stabilizes the antennal Fucα1‐2. In this mode, the antennal Galα1‐3(Fucα1‐2)Galβ1‐3 moieties of BLe^b^ interact similarly to those in the PLTL/BGB complex. The only exception occurs in the binding site between chains A and B, where Arg11 stabilizes the O2 and O3 oxygens of the Galα1‐3 moiety. The anomeric GlcNAc engages in interactions via its 2‐acetamido group, forming a hydrogen bond through O7 with Asn88, facilitated by a water molecule. Additionally, its C8 methyl group is stabilized through hydrophobic interactions with the side chains of Phe59 and Arg117. Finally, the oxygen atom O4 of Fucα1‐4 interacts with the side chain of Asp124 via a water molecule. In contrast, the newly identified noncanonical orientation of BLe^b^ was observed in the binding site between chains E and F. In this mode, Fucα1‐4 is recognized in the binding pocket in the same manner as Fucα1‐2 in the canonical orientation (Fig. 8C). However, three key interactions set this orientation apart: the anomeric oxygen of GlcNAc interacts with Arg117 via a water molecule, the Galβ1‐3 oxygen atom O6 forms a hydrogen bond with Asn88, and the Fucα1‐2 moiety establishes a water‐mediated contact with Tyr53.

The PLTL/Le^Y^ complex crystallized in the *P*4_1_2_1_2 space group, and its structure was refined to a resolution of 1.6 Å. The electron density of the oligosaccharide was well defined in only two of the three possible binding sites in ASU, while the third site (between chains A and C) remained unoccupied due to steric hindrance within the crystal. In the occupied sites, the Le^Y^ tetrasaccharide adopts an orientation similar to the canonical BLe^b^ binding site, with PLTL recognizing and stabilizing Fucα1‐2 in a manner consistent with its interaction in the PLTL/BLe^b^ complex (Fig. 8D). Additionally, the GlcNAc moiety forms a hydrogen bond with the side chain of Asn88, while the stabilization of Fucα1‐3 is mediated through water molecules interacting with the side chain of Asp124.

### Biological interactions of PLTL

**Fig. 9:**
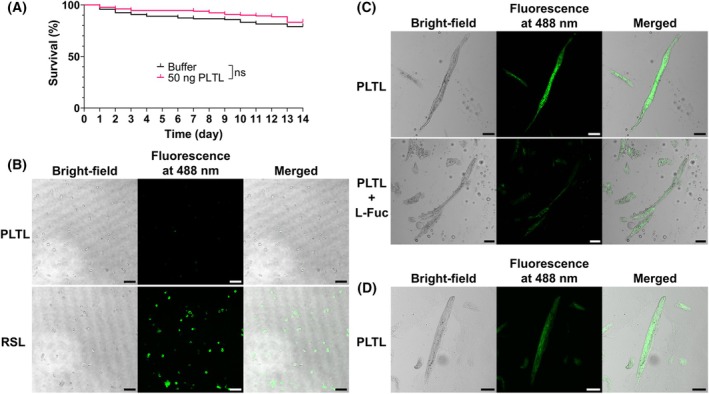
Investigating biological interactions of *Photorhabdus laumondii* tumor necrosis factor‐like lectin (PLTL). (A) Adult *D. melanogaster* were injected with PLTL, and survival was monitored daily for 14 days. No significant difference in survival was observed compared with buffer‐injected controls (Mantel‐Cox test, *P* = 0.3407, *n* = 120 adult flies per group). (B) DyLight488‐labeled PLTL did not bind to *G. mellonella* hemocytes, while the fucose‐binding lectin RSL (positive control) bound to all hemocytes. (C) PLTL binding was detected on tissues of *H. bacteriophora* infective juveniles and was abolished following pretreatment of PLTL with l‐fucose. (D) The binding of PLTL to tissues was also observed in *C. elegans*. Scale bar = 50 μm. All experiments were repeated four times; representative images are shown.

To investigate the potential biological role of PLTL, we examined its interactions with nematodes and insects—organisms naturally associated with *Photorhabdus* bacteria. Injection of PLTL into adult *Drosophila melanogaster* did not result in increased mortality over a 14‐day period compared with buffer‐injected controls (Mantel‐Cox test, *P* = 0.3407), indicating that PLTL is not toxic to insects (Fig. 9A). Furthermore, fluorescently labeled PLTL did not bind to hemocytes of *Galleria mellonella*, although the fucose‐binding lectin RSL, used as a positive control, showed strong binding to all cells (Fig. 9B). These results suggest that PLTL does not target insect tissues and is unlikely to play a direct role in *Photorhabdus* pathogenicity in insect hosts.

In contrast, PLTL bound to the tissues of the entomopathogenic nematode *H. bacteriophora*, and this interaction was specifically inhibited by preincubation with l‐fucose (Fig. 9C), indicating a fucose‐dependent binding mechanism. A similar binding pattern was observed in the free‐living nematode *Caenorhabditis elegans* (Fig. 9D), supporting the notion that PLTL recognizes conserved saccharide structures present in nematode tissues. These findings point toward a potential role for PLTL in mediating *Photorhabdus–nematode* mutualistic interactions.

## Discussion

**Fig. 10:**
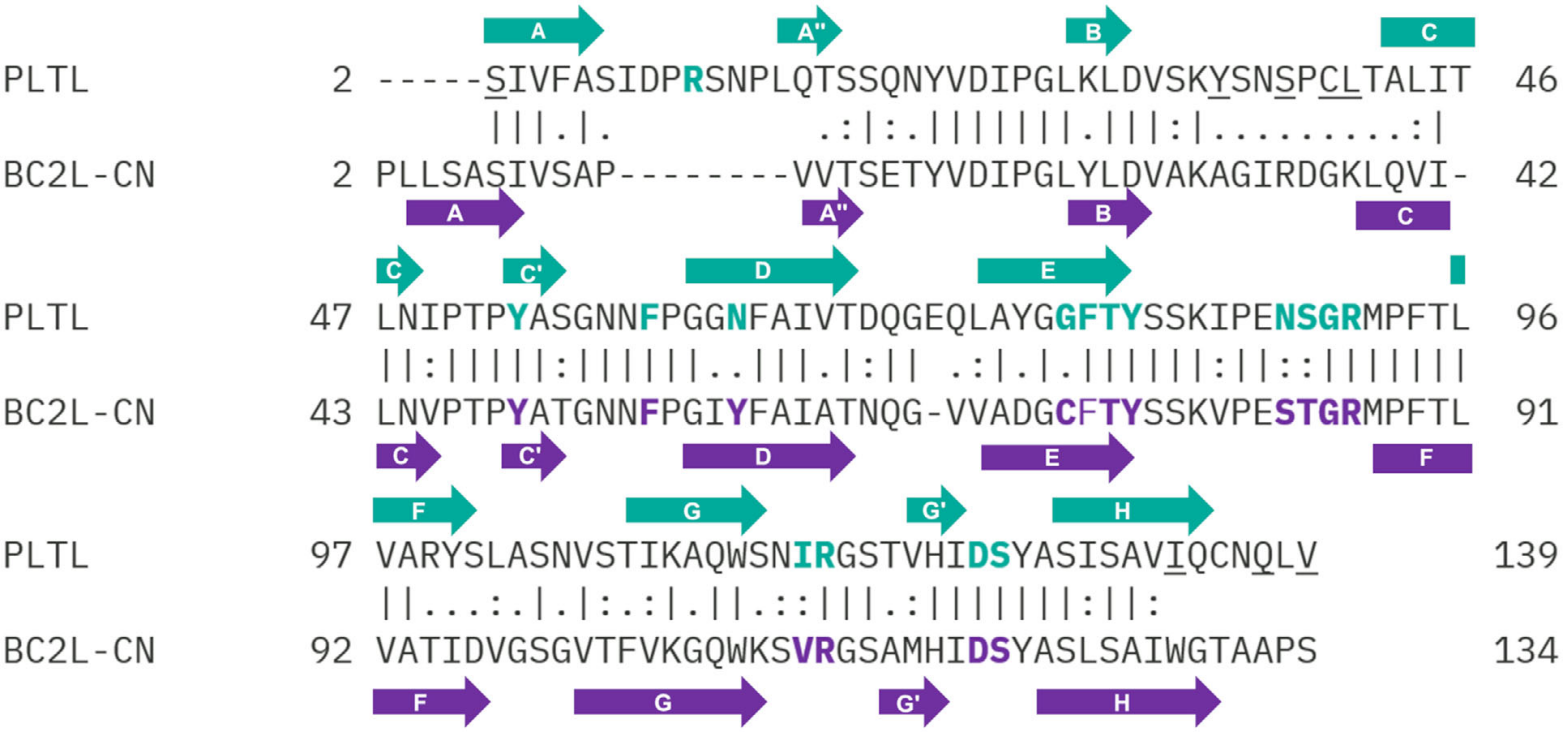
Sequence alignment of BC2L‐CN (UniProt ID: B4EH86) and *Photorhabdus laumondii* tumor necrosis factor‐like lectin (PLTL) (UniProt ID: Q7MZP1) lectins performed using EMBOSS Needle [53]. Vertical bars indicate identical residues, colons represent high amino acid similarity, and dots indicate low similarity. Full arrows mark the positions of β‐strands. Colored residues in bold form the lectin binding pockets, while underlined amino acids are involved in hexamerization of the PLTL lectin.

We have characterized a new TNF‐like lectin, PLTL, produced by the entomopathogenic bacteria *Photorhabdus laumondii*. This lectin was discovered due to its remarkable sequence identity of 52% and similarity of 65% (Fig. 10) with the BC2L‐CN lectin previously identified in the pathogenic bacterium *B. cenocepacia* [12].

Recombinant lectin PLTL is a hexamer in both solution and crystal structure, in contrast to the other known TNF‐like proteins [12, 23, 24, 25, 26], which usually adopt a trimeric structure. The hexameric structure is stabilized through disulfide bridges of opposing protomers despite being recombinantly produced in *E. coli* cytosol, which typically prevents disulfide bond formation. Oligomeric stabilization through disulfide bridges is an earlier identified feature of lectins from *Photorhabdus* spp., described in the seven‐bladed β‐propeller lectins PHL and PLL from *P. asymbiotica* [7] and *P. kayaii* [6], respectively.

**Fig. 11:**
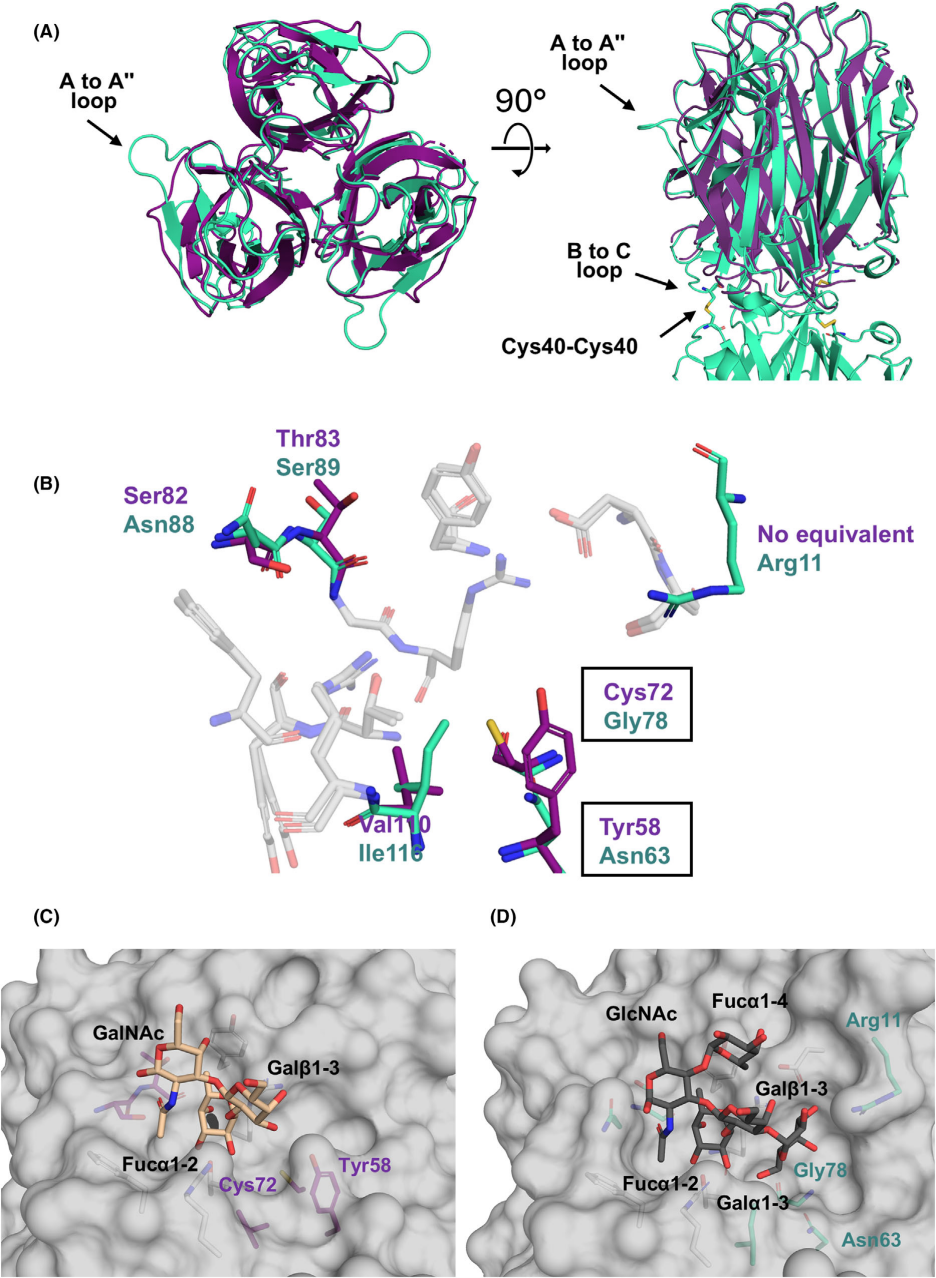
Comparative analysis of *Photorhabdus laumondii* tumor necrosis factor‐like lectin (PLTL) and BC2L‐CN. (A) Structural alignment of the TNF‐like structures of BC2L‐CN/GloboH (PDB: 6TIG, purple) and PLTL/BLe^b^ (PDB: 9IB9, green), showing an RMSD of 0.514 Å. (B) Superposition of amino acids forming the binding pockets of PLTL and BC2L‐CN. Conserved residues are depicted as white sticks, while nonconserved residues are shown in purple for BC2L‐CN and green for PLTL. Amino acids defining the binding pocket width are highlighted with rectangles. (C) Surface representation of the BC2L‐CN binding pocket with the bound GloboH antigen (beige sticks, displayed only moieties inside the binding pocket). (D) Surface representation of the PLTL binding pocket with the bound BLe^b^ antigen (gray sticks). All molecular structure images were generated using Pymol 3.0 (Schrödinger, LLC).

Structural comparison of lectin PLTL with another TNF‐like lectin, BC2L‐CN, was performed to elucidate the distinction in the binding properties of these two lectins. Upon structural alignment, TNF‐like structures of the BC2L‐CN (PDB: 6TIG) and the PLTL (PDB: 9IB9, chains A, B, C), RMSD of 0.514 Å was calculated across 272 structurally aligned Cα atoms, indicating a high degree of structural similarity. The main differences between the two structures are in the loops connecting β‐strands A and A″ and B and C. In PLTL, the A to A″ loop, which is in the proximity of the binding site, is elongated compared with BC2L‐C. Elongated B to C loops are located at the hexamerization interface and allow Cys40‐Cys40 disulfide bridge formation (Fig. 11A).

A closer look at both lectins' binding pockets explains their binding preference, as the dimensions of the lectin binding pockets predetermine the size of the glycan they natively accommodate, as discussed in [27]. Comparison of the PLTL binding site in the complex with the BLe^b^ antigen and the binding pocket of the BC2L‐CN in the complex with Globo H (Fig. 11B) shows that all amino acids interacting with Fucα1‐2 are conserved. The main difference in the lectin–saccharide interaction between PLTL and BC2L‐CN is the width of the binding pocket. BC2L‐CN contains more bulky amino acids Tyr58 and Cys72 (Fig. 11C), whereas PLTL has sterically smaller amino acids Asn63 and Gly78 in the same positions (Fig. 11D). This extra binding pocket space enables PLTL to interact with the Galα1‐3 moiety. The stabilization of the Galα1‐3 is mediated by a network of highly ordered water molecules. Notably, within the binding site between chains A and B, the Galα1‐3 moiety interacts with Arg11 on the A to A″ loop, which is significantly longer in PLTL compared with BC2L‐CN. As a result, Arg11 has no equivalent amino acid in the BC2L‐CN structure. The interaction mediated by water molecules indicates flexible binding mechanisms that may accommodate specific variations of the glycans. These findings support our observations from the hemagglutination assay and glycan array method, demonstrating PLTL's ability to bind histo‐blood group oligosaccharide A containing GalNAcα1‐3 at the glycan terminus instead of Galα1‐3.

Although both PLTL and BC2L‐CN recognize only fucosylated glycans and can be classified as fucose‐specific lectins, they exhibit very low affinity toward the l‐fucose monosaccharide. The PLTL specifically recognizes Fucα1‐2‐containing motifs and agglutinates red blood cells from all ABO groups, although the efficiency varies. These differences reflect variability in the branching patterns of human blood group A and B antigens containing GalNAcα1‐3 and Galα1‐3, respectively, emphasizing the importance of antennal saccharide identity. Glycan array analysis revealed PLTL's narrow specificity for branched glycans containing Fucα1‐2 motifs, identifying ALe^Y^, BLe^Y^, BLe^b^, and the blood group B tetrasaccharide as its preferred ligands. Consistent with these findings, SPR experiments demonstrated that PLTL's binding affinity increases with oligosaccharide complexity, showing the highest affinity for the branched BLe^b^ pentasaccharide. Interestingly, discrepancies were observed between the glycan array and SPR results for the linear Le^b^ antigen. While the glycan array did not detect Le^b^ as a ligand, SPR experiments identified it as the second‐best binding partner, likely due to the enhanced accessibility of the Fucα1‐4 moiety in solution during the SPR analysis. This accessibility is supported by the crystal structure of the PLTL/BLe^b^ complex, where Fucα1‐4 is accommodated within the binding site. Our findings highlight the distinct binding preferences of PLTL compared with BC2L‐CN, which has a strong preference for blood group O and nonbranched Lewis antigens [12, 13].

**Fig. 12:**
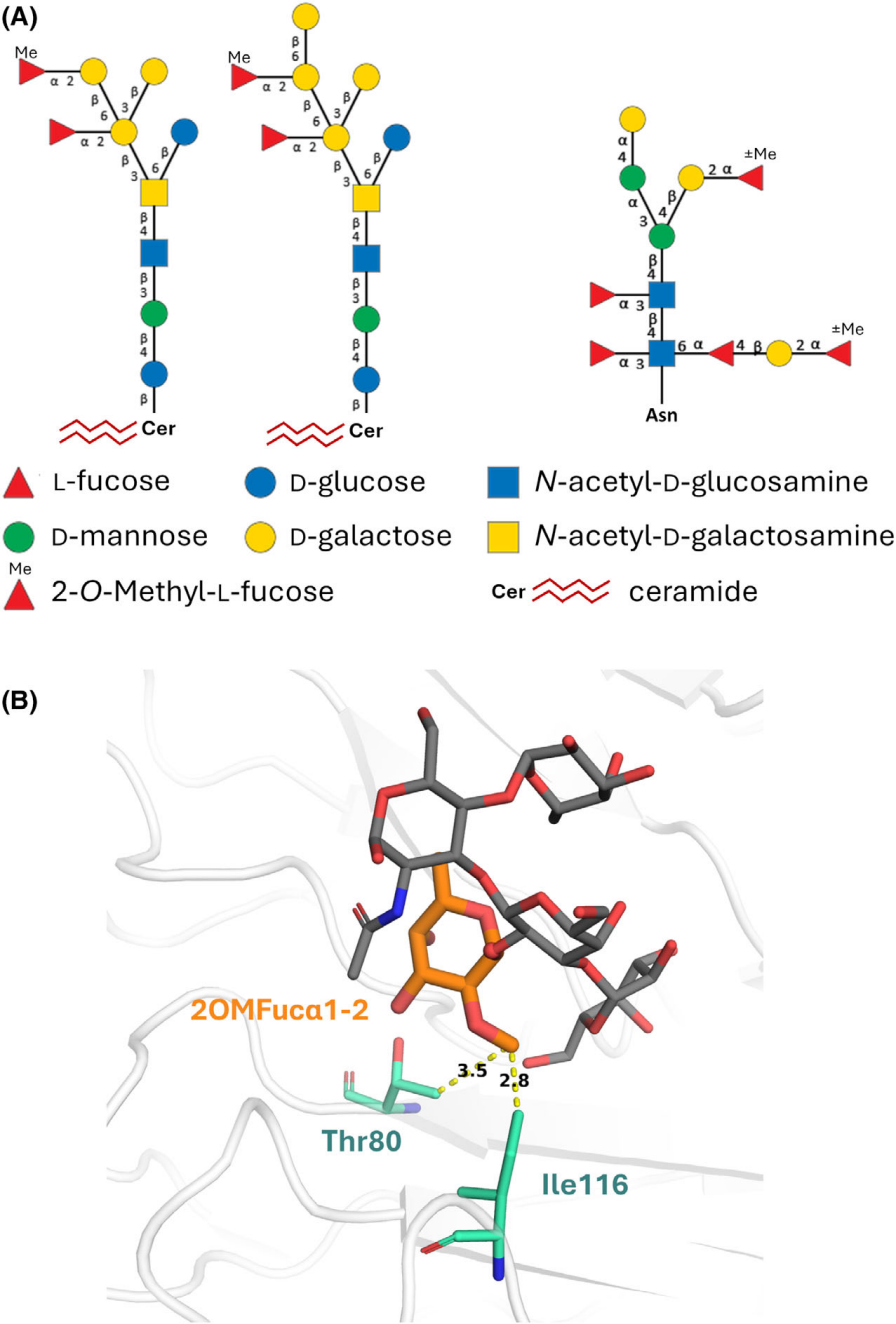
*C. elegans* glycans and possible recognition of 2‐*O*‐Methyl‐Fucose by the *Photorhabdus laumondii* tumor necrosis factor‐like lectin (PLTL) lectin. (A) Example of the *C. elegans* wild‐type glycosphingolipids and N‐glycans (modified from [54]. Glycan residues are represented according to the Symbol Nomenclature for Glycans guidelines [52]. (B) Replacement of Fucα1‐2 by 2‐*O*‐Methyl‐α‐l‐fucose (2OMFucα1‐2, orange sticks) in the structure of PLTL/BLe^b^ complex. Protein residues Thr80 and Ile116 that could stabilize the 2‐*O*‐methyl group are represented by sticks. Distances (Å) between hydrophobic groups are represented by yellow dashes. All molecular structure images were generated using Pymol 3.0 (Schrödinger, LLC).

To explore the biological role of PLTL and trace its native binding ligands, we examined its interaction with insects and nematodes. In insects, we observed neither increased *D. melanogaster* mortality after PLTL injection nor its binding to *G. mellonella* hemocytes. These findings align with PLTL's narrow specificity for Fucα1‐2 linkages, as the predominant fucosylated glycans in insects typically feature α1‐3 or α1‐6 linkages [28]. Since the glycome of the mutualistic partner *Heterorhabditis* nematode is not known, we extended our search for PLTL natural ligands to the free‐living nematode *C. elegans*, a model organism with a well‐characterized glycome. Previous *C. elegans* glycomics studies have shown that the Fucα1‐2 motif and its 2‐*O*‐methyl derivative are present in this nematode's N‐glycans and glycosphingolipids (Fig. 12A) [29, 30]. Indeed, we revealed that PLTL binds to the internal tissues of *C. elegans* and similarly binds to *H. bacteriophora*. In infective juveniles of *H. bacteriophora*, binding was characterized by a granule‐like pattern surrounding the alimentary tract along the entire body length, displaying patterns resembling intestinal granules, also known as lysosome‐related organelles (LROs) [31]. Considering PLTL's narrow glycan‐binding specificity and its binding to both tested nematodes, our results suggest that infective juveniles of *H. bacteriophora* may present Fucα1‐2 or 2‐*O*‐Methyl‐α‐*l*‐fucose glycan modifications, similar to those observed in *C. elegans*. Furthermore, fitting the 2OM‐Fuc model into the PLTL binding sites (Fig. 12B) supports the potential interaction with glycans containing this moiety.

The exact biological function of PLTL remains unclear, but our findings suggest that it plays a role in interactions with the nematode *H. bacteriophora* rather than with insect hosts. *Photorhabdus* is an obligate mutualist that provides both nutrition and developmental signals to *H. bacteriophora*, which does not harbor a diverse gut microbiota or rely on other bacteria for survival. The PLTL lectin may be involved in the maintenance of *Photorhabdus–Heterorhabditis* mutualism by facilitating binding to nematode tissues or participating in nematode defense against pathogens. Lectins in nematodes are upregulated upon pathogen exposure and play an important role in nematode defense [32]. For example, in *Caenorhabditis elegans*, lectins such as galectin LEC‐8 demonstrate the critical role of saccharide‐binding molecules in nematode biology. LEC‐8 was shown to compete for binding to glycolipids on LROs in *C. elegans*, protecting the nematode during bacterial infection [33]. PLTL may have evolved as a saccharide‐binding molecule to compensate for a function lost by *Heterorhabditis* nematodes during co‐evolution with *Photorhabdus*. This gene function loss is particularly notable in the C‐type lectin domains, which are expressed in *C. elegans* as part of its immune response to bacterial infections [34]. In fact, the genome of *H. bacteriophora* contains only nine identified C‐type‐like genes, in stark contrast to the 133 found in *C. elegans* [4].

In conclusion, PLTL stands out due to its distinctive features, including its TNF‐like hexameric structure stabilized by disulfide bridges and its unique carbohydrate specificity, characterized by a binding pocket capable of simultaneously accommodating Fucα1‐2 and Galα1‐3 moieties. In contrast to trimeric BC2L‐CN, which preferentially binds to nonbranched glycans such as blood group O antigens, PLTL shows a marked preference for branched Lewis antigens. Furthermore, PLTL interacts specifically with nematode tissues, suggesting its crucial role in the symbiotic adaptation between *P. laumondii* and *H. bacteriophora* nematodes. Further investigation of PLTL could offer deeper insights into glycoscience and provide a foundation for advancement in the study of nematode‐microbe mutualistic interactions. These distinctive properties underscore PLTL's significance in glycan recognition, highlighting its potential for applications in glycan‐targeted therapies and also the development of novel diagnostic tools.

## Materials and methods

### Production of native PLU

Bacteria *P. laumondii* ssp. *laumondii* TT0‐CCM 7076 (obtained from the Czech Collection of Microorganisms) were cultivated in minimal medium (33 mm Na_2_HPO_4_, 22 mm KH_2_PO_4_, 8.5 mm NaCl, 48 mm NH_4_Cl, 3 mm iron(III) citrate, 2 mm MgCl_2_, 0.1 mm CaCl_2_, 2% d‐glucose, pH 7.4) at 30 °C for 7 days. The culture was harvested by centrifugation (10 min, 4 °C, 12 000 ***g***) and resuspended in 20 mm Tris/HCl, 100 mm NaCl, pH 8.2 buffer. Cells were disintegrated by ultrasonic vibration and centrifuged (60 min, 4 °C, 21 000 ***g***). Soluble proteins were separated by SDS/PAGE using 14% gels stained with Coomassie Brilliant Blue R‐250 (Sigma‐Aldrich, Saint Louis, USA). The gel region corresponding to a 14–15 kDa protein size was excised, followed by in‐gel enzymatic digestion by trypsin (120 min, 40 °C) and LC–MS/MS analysis.

### Gene cloning

The sequence of hypothetical lectin PLTL was previously identified using bioinformatic tools [12] in the genome of *Photorhabdus laumondii* subsp. *laumondii STTO1* (*Taxonomy* ID: 243265, NCBI reference sequence: BX470251.1) [35].

The sequence of the *pltl* gene (NCBI reference sequence: WP_011148338.1) was codon optimized for expression in *E. coli* and synthesized by GeneArt (Thermo Fisher Scientific, Waltham, MA, USA). For cloning, the coding sequence of *pltl* was flanked with *Nde*I and *Hind*III restriction sites. The gene was cloned into the Novagen's expression vector pET25b (Merck KGaA, Darmstadt, Germany) using *Nde*I and *Hind*III restriction enzymes and T4 DNA ligase (all NEB, Ipswich, MA, USA). The sequence of the pET25b‐*pltl* construct was verified by Sanger sequencing.

### Protein production and purification

*E. coli* BL21(DE3) cells containing plasmid pET25b‐*pltl* were cultivated in an LB broth low‐salt medium with 100 μg·mL^−1^ ampicillin at 37 °C until OD_600_ reached 0.5. The gene expression was induced by 0.5 mm IPTG (isopropyl β‐*d*‐1‐thiogalactopyranoside) and performed for 18 h at 18 °C. After expression, the cells were harvested by centrifugation (15 min, 4 °C, 6600 ***g***). The pellet was resuspended in 25 mm bicine, 150 mm NaCl, pH 8.0, and stored at −20 °C for further use.

Cells were disintegrated by ultrasonication (VCX 500; Sonics & Materials, Inc., USA), and the cleared lysate was obtained by centrifugation (45 min, 4 °C, 21 000 ***g***) and subsequent filtration (pore size 0.22 μm).

The sample was heated (45 min, 85 °C), and the precipitated protein contaminants were removed by centrifugation (30 min, 4 °C, 21 000 ***g***). Heat denaturation was followed by a two‐step (NH_4_)_2_SO_4_ precipitation reaching 30% and 45% saturation. To partially remove contaminants, the appropriate amount of (NH_4_)_2_SO_4_ was added to get 30% saturation, and the mixture was gently stirred (30 min, 4 °C), followed by a collection of soluble fractions by centrifugation (15 min, 4 °C, 21 000 ***g***). Consequently, to obtain the precipitated PLTL fraction, the appropriate amount of (NH_4_)_2_SO_4_ was added to reach 45% saturation, and the mixture was gently stirred (30 min, 4 °C). Precipitated PLTL was obtained by centrifugation (15 min, 4 °C, 21 000 ***g***); the sediment was resuspended in 25 mm bicine pH 8.0 and loaded onto a Mono Q 5/50 GL column (Cytiva, Marlborough, MA, USA). The column was equilibrated with 25 mm bicine pH 8.0 buffer using an ÄKTA FPLC system (GE Healthcare, Buckinghamshire, UK). The protein was found in the flow‐through fractions. Protein purity was assessed by SDS/PAGE using 15% gels stained with Coomassie Brilliant Blue R‐250 (Sigma‐Aldrich, Saint Louis, USA).

Other studied lectins (RSL, BC2L‐CN) were produced as described previously [12, 22].

### Nano differential scanning fluorimetry

NanoDSF analysis was conducted using the Prometheus NT.48 (NanoTemper Technologies, München, Germany). PLTL samples (0.80 mg·mL^−1^) were mixed with 48 different buffer conditions [36] and loaded into nanoDSF‐grade standard capillaries (NanoTemper Technologies GmbH, München, Germany). Samples were subjected to thermal stress from 20 to 95 °C at a ramping rate of 1 °C per minute. Tryptophan fluorescence emission, following UV excitation at 280 nm, was recorded at 330 nm and 350 nm using a dual‐UV detector. Melting temperatures (*T*_m_) were calculated by the PR.ThermControl software from the 350 nm/330 nm fluorescence ratio curve.

### Analytical ultracentrifugation (AUC)

The homogeneity and oligomeric state of PLTL under different conditions were determined using Optima AUC and ProteomeLab XL‐I analytical ultracentrifuges (Beckman Coulter, Indianapolis, USA) equipped with an An50‐Ti rotor. Before the experiment, PLTL was brought to the experimental buffers by dialysis, and the buffers were used as optical references. The partial specific volume of PLTL, the solvent density, and viscosity were calculated from the amino acid sequence and buffer composition using Sednterp 3 [37]. All figures showing the results of AUC experiments were created in GUSSI 2.1.0 [38]. Sedimentation velocity experiments were carried out at 20 °C in 12 mm double‐sector centerpiece cells (Beckman Coulter, Indianapolis, USA) loaded with 425 μL of protein sample (0.62 mg·mL^−1^ PLTL in various solvents) and 425 μL of reference solutions. Data were collected using absorbance optics (280 nm) at a rotor speed of 48 000 rpm. The data were analyzed in Sedfit 18.1 [39] with the c(s) distribution model. For the regularization procedure, a confidence level of 0.95 was used.

The sedimentation equilibrium experiment was performed at 20 °C in a six‐channel centerpiece cell (Beckman Coulter, Indianapolis, USA) loaded with 100 μL of PLTL samples (0.35, 0.17, and 0.09 mg·mL^−1^ in 25 mm MES, 150 mm NaCl, pH 6.5) and 110 μL of the reference solution. Samples were gradually spun at three rotor speeds (9000, 10 800, and 19 000 rpm). After the equilibrium was achieved for each speed, scans were collected at 280 nm. To determine the molar mass of PLTL, the equilibrium data were analyzed globally in Sedphat 15.2b [40] using a noninteracting single species model with the mass conservation constraint.

### Hemagglutination assay

Experiments of hemagglutination were performed using human red blood cells (RBC) of groups A, B, and O. Blood from anonymized donors was purchased from the Transfusion and Tissue Department, The University Hospital Brno, Brno, Czech Republic. Hemagglutination experiments were performed in a 96‐well U‐bottom plate according to the procedure described previously [41]. Briefly, a 2% suspension of papain‐treated RBCs in PBS buffer (8 mm Na_2_HPO_4_, 1.5 mm KH_2_PO_4_, 137 mm NaCl, 2.7 mm KCl, pH 7.4) was mixed with equal volumes of two‐fold diluted PLTL. The mixture was incubated for 60 min at room temperature, and the results were assessed visually. As a positive control, RBCs of each blood group were mixed in a 1 : 1 ratio (v/v) with the lectin RSL to a final concentration of 250 μg·mL^−1^. For the negative controls, RBCs were mixed with equal volumes of PBS buffer.

### Ethics statement

Anonymized human blood of blood groups A, B, O, treated with sodium citrate, was purchased from the Transfusion and Tissue Department, The University Hospital Brno, Czech Republic. IRB approval for the use of blood samples was not required, as confirmed by the Ethics Committee of Masaryk University.

### Glycan array

The microarray chip (Semiotic, Moscow, Russia) [42] containing 381 saccharides in six replicates was treated with 25 mm bicine, 150 mm NaCl, 0.1% Tween‐20, pH 8.0 buffer for 15 min. The DyLight488‐labeled PLTL (0.31 μg·mL^−1^) in buffer 25 mm bicine, 150 mm NaCl, pH 8.0 was applied on the chip surface and incubated at 37 °C for 1 h. After the incubation, the chip surface was washed with 25 mm bicine, 150 mm NaCl, 0.5% Tween‐20, pH 8.0, and deionized water. The detection of bound protein was carried out at 488 nm using an InnoScan1100 AL (Innopsys, Carbonne, France). The data were analyzed with the Mapix 8.2.2 software and an online chip converter (Semiotik, https://rakitko.shinyapps.io/semiotik).

### Surface plasmon resonance

SPR experiments were conducted in a Biacore S200 instrument (Cytiva, Marlborough, MA, USA) at 25 °C. The sensor chip CM5 (Cytiva, Marlborough, MA, USA) was activated by a 0.1 M N‐ethyl‐*N*‐(3‐dimethylaminopropyl)‐carbodiimide/0.4 M *N*‐hydroxysuccinimide solution. The reference channel was consequently blocked by 1 M ethanolamine, pH 8.0. Studied lectins were immobilized on measuring channels by the injection of PLTL (50 μg·mL^−1^), BC2L‐CN (50 μg·mL^−1^), and RSL (30 μg·mL^−1^) in 10 mm sodium acetate, pH 5.0, and consequently blocked by 1 M ethanolamine, pH 8.0. Measurements were carried out in 10 mm HEPES, 150 mm NaCl, 0.05% Tween 20, pH 7.5, in all channels simultaneously, using a flow rate of 10 μL·min^−1^ with an association time of 120 s and a dissociation time of 180 s. Due to the rapid association and dissociation kinetics observed for all ligands, the steady‐state model was utilized for *K*_D_ calculation. Data analysis was conducted using the Biacore S200 Evaluation software 1.1.1, employing the one‐site binding model.

### Crystallization and data collection

The structure of the PLTL lectin apo form and its complexes with oligosaccharides BGB, Le^Y^, and BLe^b^ was determined via protein X‐ray crystallography. The protein sample (6.5 mg·mL^−1^ PLTL in 20 mm bicine, 5 mm TCEP, pH 8.0) was used for the initial screening of crystallization conditions using the sitting‐drop vapor diffusion method at 20 °C. Initial hits from commercial screening kits (Qiagen, Hilden, Germany) were further optimized and used for subsequent co‐crystallization of the PLTL complexes with 4 mm ligands. The best diffracting crystals were obtained under the following conditions: 0.1 M trisodium citrate, 3.5 M sodium chloride, pH 7.5 (apo‐PLTL); 0.1 M trisodium citrate, 17% (w/v) PEG1500, pH 4.4 (PLTL/BGB complex); 0.1 M trisodium citrate, 17% (w/v) PEG1500, pH 4.5 (PLTL/Le^Y^ complex); and 0.1 M trisodium citrate, 15% (w/v) PEG1500, pH 5.9 (PLTL/BLe^b^ complex). Protein crystals were cryoprotected for 10 s in a 1 : 1 mixture of mother liquors and 100% MPD and vitrified in liquid nitrogen. Diffraction data were collected at 100 K at the synchrotron BESSY II beamline 14.1 [43] (apo‐PLTL and PLTL/BGB) and at PETRA III, beamline P13 [44] (PLTL/Le^Y^ and PLTL/BLe^b^) using mxCube v2 [45].

### Structure determination

Data processing was performed using autoPROC [46], and the data were merged and scaled using Scala 1.12.14 [47]. The BC2L‐CN model (PDB: 2WQ4) coordinates were used as the initial model for the molecular replacement of the PLTL apo form using PHASER 2.8.3 [48]. The resulting model was consequently used for phase calculation of all models with ligands. Structural refinement was conducted in REFMAC5 5.8.0405 [49] and alternated with manual model building and correction in COOT 0.9.8.93 [50]. Cross‐validation analysis was performed using 5% of the observations. Ligands were assigned based on Fo‐Fc electron density maps with peak heights exceeding 3*σ*. The final models were validated in the PDBe validation server (https://validate‐rcsb‐1.wwpdb.org/) and deposited with PDB IDs: 9IB6, 9IB7, 9IB8, and 9IB9. The refinement statistics are given in Table 1. The 3D models and ligand restraint files were generated using the program JLigand [51].

### Biological assays of PLTL interactions

To assess the potential toxic effects of PLTL on insects, 5‐day‐old adult *Drosophila melanogaster* were injected intra‐thoracically with 50 nL of 1 mg·mL^−1^ PLTL (corresponding to 50 ng per fly) using the Nanoject III nanoinjector (Drummond Scientific, Pennsylvania, USA). Control flies received an equivalent volume of 20 mm bicine, 150 mm NaCl, pH 8.0 buffer. For each treatment, four groups consisting of 15 males and 15 females were injected. Mortality was recorded daily over a 14‐day observation period.

To evaluate the interaction of PLTL with insect hemocytes, hemolymph was collected from *G. mellonella* larvae as previously described [7]. Hemocytes were allowed to adhere to glass slides for 10 min at room temperature. After incubation, hemocytes were fixed with 4% formaldehyde, pH 6.9 (Merck, Darmstadt, Germany) for 10 min at room temperature, followed by three washes in PBS buffer for 5 min each. Hemocytes were then treated for 10 min with DyLight488‐labeled PLTL or RSL [22] lectins (both 0.1 mg·mL^−1^), washed 3 × 5 min in PBS buffer, and mounted in Dako medium (Sigma‐Aldrich, Saint Louis, USA) for confocal microscopy using Leica SP8 (Leica, Wetzlar, Germany).

The interaction between DyLight488‐labeled PLTL and nematodes was tested on infective juveniles of the entomopathogenic nematode *Heterorhabditis bacteriophora* H221 (isolated from Pouzdřany, Czech Republic, and kept in the laboratory collection at Masaryk University, Brno) and free‐living nematode *Caenorhabditis elegans* (reared on agar plates with *Escherichia coli* OP50). The microsections were prepared from both nematode species after washing in tap water, filter‐sterilized using a 0.22 μm cellulose acetate membrane (Whatman, Maidstone, United Kingdom), embedded in Tissue‐Tek^®^ O.C.T. Compound (Sakura Finetek, CA, USA), and frozen at −80 °C. The prepared blocks with frozen nematodes were cut using a cryostat, Leica CM1850, into 12 μm‐thick sections. The sections were collected onto SuperFrost Plus slides (Thermo Fisher Scientific, Waltham, MA, USA), fixed for 10 min in 4% formaldehyde, pH 6.9 (Merck, Darmstadt, Germany), and then washed 3 × 5 min in PBS buffer. Following the wash, the sections were incubated in 1% BSA for 1 h at room temperature and then treated with DyLight488‐labeled PLTL (0.1 mg·mL^−1^) overnight in the dark at 4 °C. Saccharide inhibition was conducted by preincubating PLTL with 0.25 M l‐fucose prior to application to the tissue samples. After incubation, the nematode sections were washed 3 × 5 min in PBS buffer, mounted in Dako medium (Sigma‐Aldrich, Saint Louis, USA), and observed using an SP8 laser scanning confocal microscope (Leica, Wetzlar, Germany).

## Conflict of interest

The authors declare no conflict of interest.

## Author contributions

MW supervised the work. MW, FM, PD, and JH contributed to planning experiments. FM, PD, JK, LF, MK, and PS performed experiments. FM, PD, and JK analyzed data. FM and PD prepared figures and wrote the original draft. All authors have reviewed and edited the manuscript and approved the published version of the manuscript.

## Supporting information




**Fig. S1.** SPR sensorgrams displaying saccharides binding to lectin PLTL.
**Fig. S2.** SPR sensorgrams displaying saccharides binding to lectin BC2L‐CN.
**Fig. S3.** SPR sensorgrams displaying saccharides binding to lectin RSL.
**Table S1.** Summary of the interaction of PLTL with oligosaccharides.

## Data Availability

The structural data that support these findings are openly available in the wwPDB at https://doi.org/10.2210/pdb9IB6/pdb (apo‐PLTL), https://doi.org/10.2210/pdb9IB7/pdb (PLTL/BGB complex), https://doi.org/10.2210/pdb9IB8/pdb (PLTL/Le^Y^ complex), and https://doi.org/10.2210/pdb9IB9/pdb (PLTL/BLe^b^ complex). All other data that support the findings of this study are available in the figures and tables in the manuscript and in the Supporting Information of this article.
