# Supplementary material for: Structural and functional characterization of the newly identified Photorhabdus laumondii tumor necrosis factor‐like lectin
Source: FEBS J. 2025 Oct 17;293(4):1168–90. doi: 10.1111/febs.70293 (PMC12914763; doi:10.1111/febs.70293)
Supplement: Supplementary file 1 — Fig. S1. SPR sensorgrams displaying saccharides binding to lectin PLTL. Fig. S2. SPR sensorgrams displaying saccharides binding to lectin BC2L‐CN. Fig. S3. SPR sensorgrams displaying saccharides binding to lectin RSL. Table S1. Summary of the interaction of PLTL with oligosaccharides. [file FEBS-293-1168-s001.pdf]

Supporting Information

Title: Structural and functional characterization of the newly identified Photorhabdus laumondii tumor necrosis factor-like lectin

Authors: Filip Melicher[1,2], Pavel Dobeš[2,3], Jan Komárek[1,2], Lukáš Faltinek[4], Marek Korsák[1,2], Petra Sýkorová[1], Josef Houser[1,2], Michaela Wimmerová[1,2,4] *

Affiliations: [1]*Central European Institute of Technology (CEITEC), Masaryk University, Brno, Czech Republic*

[2]*National Centre for Biomolecular Research, Faculty of Science, Masaryk University, Brno, Czech Republic*

[3]*Department of Experimental Biology, Faculty of Science, Masaryk University, Brno, Czech Republic*

[4]*Department of Biochemistry, Faculty of Science, Masaryk University, Brno, Czech Republic*

Running Title: Novel TNF-like lectin PLTL from *P. laumondii*

Content:

Figure S1: SPR sensorgrams displaying saccharides binding to lectin PLTL.

Figure S2: SPR sensorgrams displaying saccharides binding to lectin BC2L-CN.

Figure S3: SPR sensorgrams displaying saccharides binding to lectin RSL.

Table S1: Summary of the interaction of PLTL with oligosaccharides.

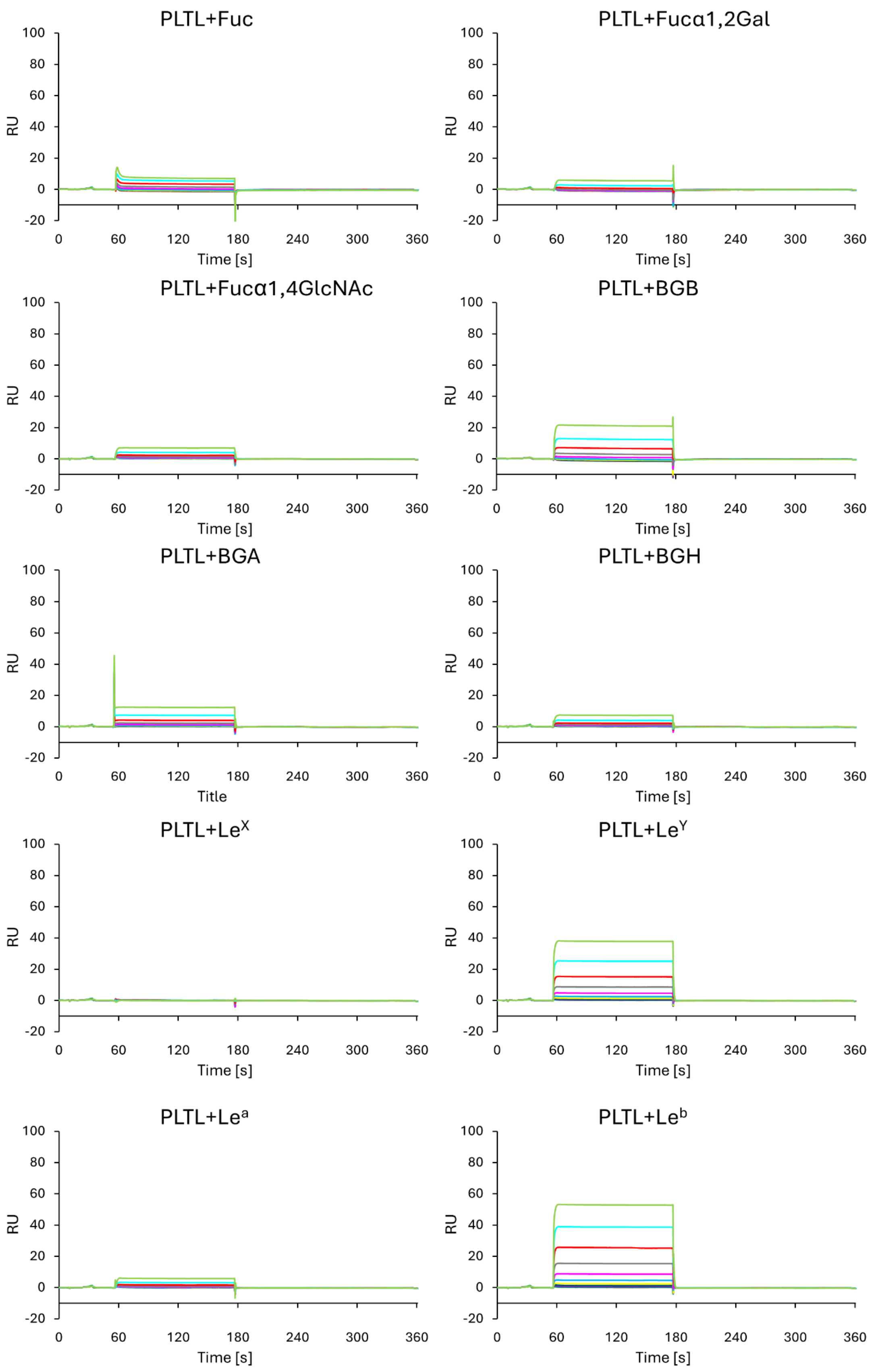

Figure S1: SPR sensorgrams displaying saccharides binding to lectins PLTL.

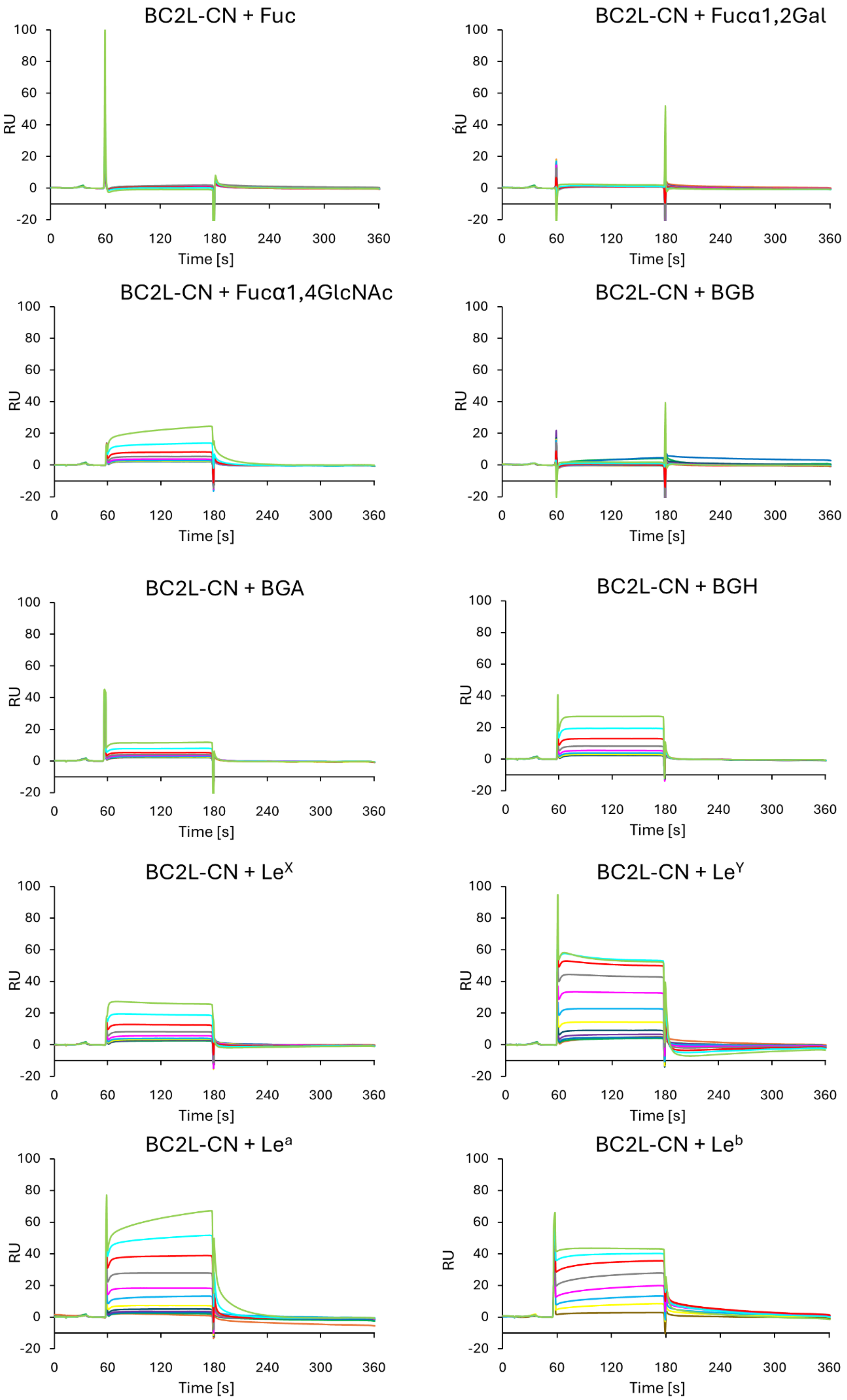

Figure S2: SPR sensorgrams displaying saccharides binding to lectins BC2L-CN.

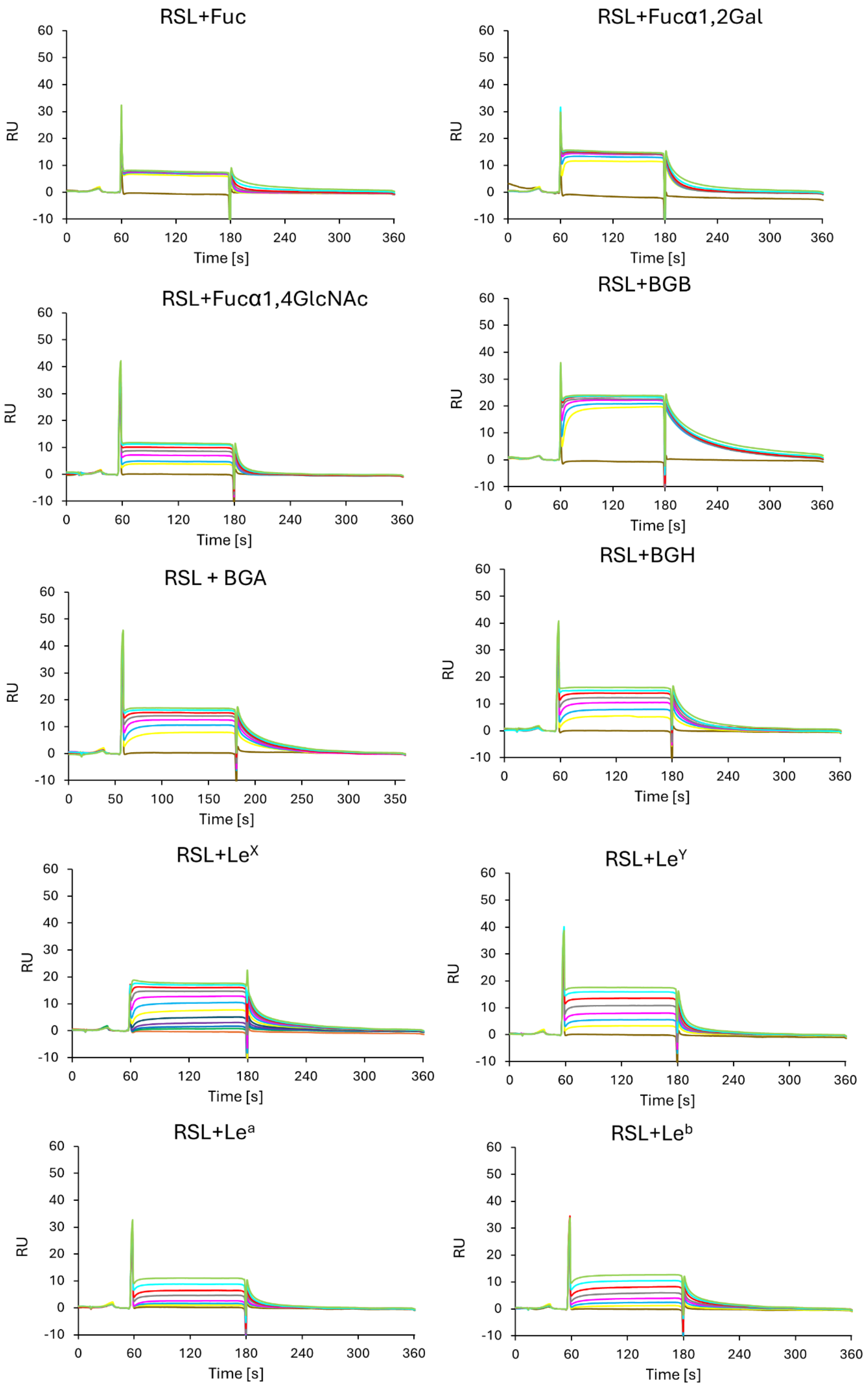

Figure S3: SPR sensorgrams displaying saccharides binding to lectins RSL.

Table S1: Summary of the interaction of PLTL with oligosaccharides. For ligands with multiple copies in ASU, mean distances and standard deviations were calculated from the distances in each protomer.

PLTL/Bleb canon		
Fucα12		
	Protein atom or water	Distance (Å)
O2	Arg117*NH1	2.94 ± 0.01
	Arg117*NH2	3.02 ± 0.02
O3	Thr80*OG	2.68 ± 0.01
	Arg117*NH2	3.01 ± 0.02
	HOH1→ Tyr81*O	2.64 ± 0.01→2.76 ± 0.03
	HOH1→Asn88*NH2	2.64 ± 0.01→2.81 ± 0.03
O4	Ser89*O	2.75 ± 0.02
	Arg91*NE	3.00 ± 0.03
O5	Arg91*NE	3.17 ± 0.01
	Arg91*NH2	2.84 ± 0.02
C6	Tyr53	Hydrophobic interaction
Galα13		
O2	Arg11*NH2	3.20
	HOH2→Asp124*O	3.01 ± 0.07→2.79 ± 0.04
O3	HOH2→Asp124*O	2.87 ± 0.01→2.75 ± 0.1
	Asp124*OD1	2.80 ± 0.08
O4	HOH3→Asn63*O	2.79 ± 0.05→2.84 ± 0.04
O5	HOH4→Asn63*ND2	2.83 ± 0.09→3.09 ± 0.07
O6	HOH5→Phe79*O	2.74 ± 0.01→3.07 ± 0.02
	HOH5→Asn63*OD1	2.74 ± 0.01→2.79 ± 0.01
GlcNAc		
O7	HOH6→Asn88*OD1	2.8 ± 0.03→2.8 ± 0.19
C8	Phe59<br>Arg117	Hydrophobic interaction
Fucα14		
O4	HOH→Asp124*OD2	2.64 ± 0.05→2.70 ± 0.03

PLTL/Bleb non cannon		
Fucα14		
	Protein atom or water	Distance (Å)
O2	Arg117*NH1	2.94
	Arg117*NH2	3.07
O3	Thr80*OG	2.69
	Arg117*NH2	3.14
	HOH1→Tyr81*O	2.67→2.79
	HOH1→Asn88*NH2	2.67→2.99
O4	Ser89*O	2.65
	Arg91*NE	2.97
O5	Arg91*NE	3.19
	Arg91*NH2	2.88
C6	Tyr53	Hydrophobic interaction
GlcNAc		
O3	HOH2→Arg117*NH1	3.00→2.93
Galβ13		
O6	Asn88*OD	2.76
Fuc12		
O4	HOH3→Tyr53*OH	2.94→2.63
O5	HOH3→Tyr53*OH	2.91→2.63
Galα13		
O2	HOH4→Ser89*N	2.71→3.01

PLTL/BGB		
Fucα12		
	Protein atom or water	Distance (Å)
O2	Arg117*NH1	2.95 ± 0.06
	Arg117*NH2	3.04 ± 0.03
O3	Thr80*OG	2.61 ± 0.07
	Arg117*NH2	3.15 ± 0.22
	HOH1→ Tyr81*O	2.63 ± 0.01→2.67 ± 0.02
	HOH1→Asn88*NH2	2.63 ± 0.01→2.78 ± 0.04
O4	Ser89*O	2.66 ± 0.03
	Arg91*NE	2.97 ± 0.07
O5	Arg91*NE	3.11 ± 0.07
	Arg91*NH2	2.84 ± 0.03
C6	Tyr53	Hydrophobic interaction
Galα13		
O2	Arg11*NH2	-
	HOH2→Asp124*O	3.06 ± 0.02→2.79 ± 0.04
O3	HOH2→Asp124*O	2.87 ± 0.01→2.75 ± 0.10
	Asp124*OD1	2.83 ± 0.11
O4	HOH3→Asn63*O	2.86 ± 0.04→2.70 ± 0.07
O5		
O6	HOH4→Phe79*O	2.80 ± 0.02→3.09 ± 0.02
	HOH4→Asn63*OD1	2.80 ± 0.02→2.80 ± 0.07

PLTL/LeY		
Fucα12		
	Protein atom or water	Distance (Å)
O2	Arg117*NH1	2.96 ± 0.01
	Arg117*NH2	3.03 ± 0.01
O3	Thr80*OG	2.64 ± 0.01
	Arg117*NH2	3.15 ± 0.08
	HOH1→Tyr81*O	2.65 ± 0.03→2.76 ± 0.01
	HOH1→Asn88*NH2	2.65 ± 0.03→2.71 ± 0.03
O4	Ser89*O	2.74 ± 0.04
	Arg91*NE	3.01 ± 0.04
O5	Arg91*NE	3.15 ± 0.04
	Arg91*NH2	2.84 ± 0.01
C6	Tyr53	Hydrophobic interaction
Galβ13		
O3	HOH2→Asp124*O	2.90→3.05
O4	HOH2→Asp124*O	2.90→3.09
Fucα14		
O6	HOH2→Asp124*OD2	2.77→2.62
